# Efficient mitochondrial biogenesis drives incomplete penetrance in Leber’s hereditary optic neuropathy

**DOI:** 10.1093/brain/awt343

**Published:** 2013-12-24

**Authors:** Carla Giordano, Luisa Iommarini, Luca Giordano, Alessandra Maresca, Annalinda Pisano, Maria Lucia Valentino, Leonardo Caporali, Rocco Liguori, Stefania Deceglie, Marina Roberti, Francesca Fanelli, Flavio Fracasso, Fred N. Ross-Cisneros, Pio D’Adamo, Gavin Hudson, Angela Pyle, Patrick Yu-Wai-Man, Patrick F. Chinnery, Massimo Zeviani, Solange R. Salomao, Adriana Berezovsky, Rubens Belfort, Dora Fix Ventura, Milton Moraes, Milton Moraes Filho, Piero Barboni, Federico Sadun, Annamaria De Negri, Alfredo A. Sadun, Andrea Tancredi, Massimiliano Mancini, Giulia d’Amati, Paola Loguercio Polosa, Palmiro Cantatore, Valerio Carelli

**Affiliations:** 1 Department of Radiology, Oncology and Pathology, Sapienza, University of Rome, Rome, Italy; 2 Department of Biomedical and NeuroMotor Sciences (DIBINEM), University of Bologna, Bologna, Italy; 3 Department of Biosciences, Biotechnologies and Biopharmaceutics, University of Bari, Bari, Italy; 4 IRCCS Istituto delle Scienze Neurologiche di Bologna, Bellaria Hospital, Bologna, Italy; 5 Departments of Ophthalmology and Neurosurgery, Keck School of Medicine at USC, Los Angeles, CA, USA; 6 Medical Genetics, Department of Reproductive Sciences, Development and Public Health; 7 IRCCS-Burlo Garofolo Children Hospital, University of Trieste, Trieste, Italy; 8 Institute of Genetic Medicine, Newcastle University, Newcastle upon Tyne, UK; 9 Unit of Molecular Neurogenetics, Fondazione Istituto Neurologico “Carlo Besta” - IRCCS, Milano, Italy; 10 MRC-Mitochondrial Biology Unit, Cambridge, UK; 11 Department of Ophthalmology, Federal University of Sao Paulo – UNIFESP, Sao Paulo, Brazil; 12 Department of Experimental Psychology, Institute of Psychology, University of Sao Paulo, Sao Paulo, Brazil; 13 Studio Oculistico d’Azeglio, Bologna, Italy; 14 Ospedale San Giovanni Evangelista, Tivoli, Italy; 15 Azienda Ospedaliera San Camillo-Forlanini, Roma, Italy; 16 Dipartimento di Metodi e Modelli per l’Economia la Finanza e il Territorio, Sapienza, Università di Roma, Roma, Italy; 17 Department of Molecular Medicine, Sapienza, University of Rome

**Keywords:** LHON penetrance, mitochondrial biogenesis, mtDNA copy number

## Abstract

Leber’s hereditary optic neuropathy is a maternally inherited blinding disease caused as a result of homoplasmic point mutations in complex I subunit genes of mitochondrial DNA. It is characterized by incomplete penetrance, as only some mutation carriers become affected. Thus, the mitochondrial DNA mutation is necessary but not sufficient to cause optic neuropathy. Environmental triggers and genetic modifying factors have been considered to explain its variable penetrance. We measured the mitochondrial DNA copy number and mitochondrial mass indicators in blood cells from affected and carrier individuals, screening three large pedigrees and 39 independently collected smaller families with Leber’s hereditary optic neuropathy, as well as muscle biopsies and cells isolated by laser capturing from post-mortem specimens of retina and optic nerves, the latter being the disease targets. We show that unaffected mutation carriers have a significantly higher mitochondrial DNA copy number and mitochondrial mass compared with their affected relatives and control individuals. Comparative studies of fibroblasts from affected, carriers and controls, under different paradigms of metabolic demand, show that carriers display the highest capacity for activating mitochondrial biogenesis. Therefore we postulate that the increased mitochondrial biogenesis in carriers may overcome some of the pathogenic effect of mitochondrial DNA mutations. Screening of a few selected genetic variants in candidate genes involved in mitochondrial biogenesis failed to reveal any significant association. Our study provides a valuable mechanism to explain variability of penetrance in Leber’s hereditary optic neuropathy and clues for high throughput genetic screening to identify the nuclear modifying gene(s), opening an avenue to develop predictive genetic tests on disease risk and therapeutic strategies.

## Abstract (precis)

The mechanisms of incomplete penetrance in Leber’s hereditary optic neuropathy are elusive. Giordano *et al*. show that mitochondrial DNA content and mitochondrial mass are both increased in tissues and cells from unaffected mutation carriers relative to affected relatives and control individuals. Upregulation of mitochondrial biogenesis may represent a therapeutic target.

## Introduction

Leber’s hereditary optic neuropathy (LHON) is the prototypic mitochondrial optic neuropathy and the most frequent mitochondrial disease (Chinnery *et al.*, 2000; Carelli *et al.*, 2004). The primary role for mitochondrial aetiology is revealed by maternal inheritance and association with specific mitochondrial DNA point mutations affecting complex I subunit genes *MT-ND4* (m.11778G>A), *MT-ND1* (m.3460G>A), and *MT-ND6* (m.14484T>C) (Newman, 2005). The classical presentation of LHON includes the rapid loss of central vision, predominantly in young males, with the establishment of optic atrophy ∼1 year after onset (Newman, 2005; Barboni *et al.*, 2010). In most cases, pathogenic LHON mutations are homoplasmic (100% mutant mitochondrial DNA) in all maternally-related individuals, but only a subset of them will express the disease. Thus, the mitochondrial DNA mutation is necessary but not sufficient to cause optic neuropathy and disease penetrance may vary in different families with the same mutation, and even within the same family in different branches (Howell and Mackey, 1998; Carelli *et al.*, 2003). Unexplained questions regarding LHON include the incomplete penetrance, the male prevalence and the tissue-specific targeting of retinal ganglion cells. The first two issues might be addressed by the existence of modifying genes in the nuclear genome. A leading hypothesis for male prevalence suggests the involvement of chromosome X and different loci were reported by linkage and association studies in LHON families, but no significant variants have been identified to date (Hudson *et al.*, 2005; Shankar *et al.*, 2008; Ji *et al.*, 2010). A genome-wide scan of Asian LHON families suggested the existence of multiple loci, finding a significant association with two single nucleotide polymorphisms (SNPs) in the presenilin-associated rhomboid-like (*PARL*) gene (Phasukkijwatana *et al.*, 2010). However, a subsequent study on a large cohort of patients with LHON from China failed to reproduce this association (Zhang *et al.*, 2010).

A compensatory strategy to mitochondrial dysfunction commonly observed in mitochondrial diseases is the increase of mitochondrial biogenesis, as exemplified by the massive proliferation of mitochondria in skeletal muscle fibres (DiMauro and Schon, 2003). The ragged-red fibres have been reproduced in mouse by disrupting the expression of *TFAM* and the increased mitochondrial mass partly compensated for the reduced function of the respiratory chain by maintaining overall ATP production in skeletal muscle (Wredenberg *et al.*, 2002). Thus, within certain limits, an increase of mitochondrial mass can be a successful compensatory strategy. In skeletal muscle from patients with LHON a relative increase in mitochondrial mass is indicated by subsarcolemmal enhancement of succinic dehydrogenase staining as a result of parcellar accumulations of mitochondria (Larsson *et al.*, 1991; Iommarini *et al.*, 2012). Increases of the matrix enzyme citrate synthase and succinic dehydrogenase activities, both good indicators of mitochondrial mass, have also been described in LHON (Larsson *et al.*, 1991; Yen *et al.*, 1996). A few small studies reported that there was an increase in mitochondrial DNA content in blood cells from patients with LHON (Yen *et al.*, 2002; Iommarini *et al.*, 2012), as well as in unaffected mutation carriers (Nishioka *et al.*, 2004), compared with controls, suggesting the activation of mitochondrial biogenesis. Interestingly, the mitochondrial DNA content was slightly higher in carriers. Furthermore, anti-retroviral therapy, which reduces mitochondrial DNA content, apparently triggered optic neurpathy in a few cases of patients with LHON infected with HIV (Mackey *et al.*, 2003). Finally, studies on neuronal NT2 cybrids carrying the homoplasmic m.11778G>A mutation showed a pathological phenotype with increased oxidative stress only after neuronal differentiation, which remarkably reduced by 3-fold the mitochondrial DNA:nuclear DNA ratio (Wong *et al.*, 2002). Thus, we postulate that mitochondrial DNA copy number per cell and mitochondrial biogenesis may be important factors in LHON, possibly involved in protecting from or promoting the disease process.

In the present study, we show that a higher mitochondrial DNA content and increased mitochondrial biogenesis in multiple tissues differentiates the unaffected mutation carriers from LHON affected patients and control subjects. This observation provides a plausible explanation for incomplete penetrance in LHON and has broad implications for therapy in LHON and other disorders.

## Materials and methods

The procedures for *ex vivo* collection and use of human tissues for this study were approved by the Ethics Committee of the St. Orsola-Malpighi Polyclinic (University of Bologna) and the Sao Paulo Hospital (Federal University of Sao Paulo). The procedures for collection and use of human post-mortem tissues were approved by the University of Southern California Ethics Committee. All samples were obtained from donors or their families having provided written informed consent according to the Declaration of Helsinki.

Detailed experimental procedures are available in the Supplementary material.

### Leber’s hereditary optic neuropathy pedigrees and case series

We first investigated three large LHON pedigrees harbouring the m.11778G>A homoplasmic mutation on a haplogroup J background (Table 1 and Supplementary Figs 1–3). Family 1 is a previously reported large maternal lineage of Italian ancestry, now mostly living in Brazil (SOA-BR family) (Sadun *et al.*, 2003, 2004, 2006; Ventura *et al.*, 2005; Carelli *et al.*, 2006; Shankar *et al.*, 2008; Ramos Cdo *et al.*, 2009; La Morgia *et al.*, 2010; Pan *et al.*, 2012). We had available peripheral blood from 25 affected and 38 unaffected mutation carriers from this pedigree. As controls (*n = *54) we used the spouses from the same family, which do not belong to the mutant maternal lineage (off-pedigree). Family 2 is a previously reported (Carelli *et al.*, 2006) Italian family composed of 21 individuals (eight affected, eight carriers and five off-pedigree). Family 3 is an Irish-American pedigree composed of 17 individuals (two affected, 14 carriers and one off-pedigree).

**Table 1:** Demographic data of the three m.11778G>A LHON families

ID (reference)	Ethnic origin	Affected	Mean age (range)	Carriers	Mean age (range)	Controls	Mean age (range)
Family 1 (Carelli et al., 2006)	Italian-Brazilian	22 M	43 (15–84)	12 M	47 (37–79)	28 M	46 (21–75)
		3 F	63 (59–67)	26 F	48 (35–85)	26 F	44 (23–79)
Family 2 (Carelli *et al.*, 2006))	Italian	6 M	39 (21–64)	1 M	47	3 M	32 (31–33)
		2 F	46 (25–68)	7 F	51 (36–67)	2 F	28 (27–29)
Family 3	Irish-American	2 M	n.a.	7 M	n.a.	1 M	n.a.
		–	–	7 F	n.a.	–	–

n.a. = not available; F = female; M = male.

Subsequently, we studied all the available individuals from 39 unrelated Italian LHON pedigrees (64 affected and 68 carriers) harbouring one of the three common LHON mutations: m.11778/G>A/*MT-ND4*, m.3460G>A/*MT-ND1* and m.14484T>C/*MT-ND6* mutations (Table 2). A further independently-collected cohort (100 affected and 100 carriers harbouring the same common mutations) from Northern Europe was used only for the association studies investigating candidate genetic variants in genes involved in mitochondrial biogenesis.

**Table 2:** Demographic data of the 39 LHON families

Genotype	Number of Families	Affected	Mean age (range)	Carriers	Mean age (range)
m.11778G>A	24	28 M	32 (9–79)	12 M	52 (36–75)
		9 F	40 (11–65)	33 F	52 (36–73)
m.3460G>A	10	9 M	33 (8–63)	3 M	44 (36–55)
		10 F	42 (10–80)	12 F	54 (36–82)
m.14484T>C	5	6 M	29 (15–58)	–	–
		2 F	36 (35–38)	8 F	56 (36–82)

F = female; M = male.

For all cases, we assessed only unaffected carriers ≥35 years of age, to minimize the possibility that these individuals could become affected in the future.

### Ophthalmological assessments in carriers from Family 1 (SOA-BR)

The ophthalmological data used for correlative analysis with mitochondrial DNA content were previously reported assessments of ocular fundus features (Sadun *et al.*, 2004), contrast sensitivity functions (Ventura *et al.*, 2005), and optical coherence tomography measurements of retinal nerve fibre layer (RNFL) thickness and optic disc size (Ramos *et al.*, 2009), carried out in carriers from Family 1. The anatomical measures of optical coherence tomography analyses considered in this study are the RNFL thickness around the optic nerve head in the temporal, superior, nasal and inferior quadrants (QUADRANT T, S, N, I), as well as the optic disc area and the average, minimum, and six sectorial (TEMPSUP, SUP, NASSUP, NASINF, INF and TEMPINF) RNFL thicknesses measured from the annulus centred on the fovea.

### Muscle biopsies

Vastus lateralis or tibialis anterior muscle biopsies were performed by open surgery, under local anaesthesia and after informed consent of the patient. Muscle specimens were frozen in cooled isopentane and stored in liquid nitrogen for histological and histoenzymatic analysis including succinic dehydrogenase activity and adenosine triphosphatase (ATPase) staining according to standard protocols. Analysis of the distribution of type I and II fibres was performed by counting them with light microscopy on sections from tibialis anterioris stained with ATPase*.* At least 200 fibres (from 200 to 700) were counted for each biopsy.

### Optic nerve and retina studies

We examined formalin-fixed, paraffin embedded ocular post-mortem specimens as follows: sagittal histological serial sections of optic nerves from four control male subjects (left and right eyes; age range 60–80; mean 72); retrobulbar histological serial cross-sections of optic nerves from six control subjects (left and right eyes; four males, age range 58–74, mean 66); two LHON-affected eyes (both sagittal and cross-sections) from a 59-year-old male subject bearing the m.11778G>A mutation; two LHON-unaffected carrier eyes (both sagittal and cross-sections) from an 83-year-old female bearing the m.11778G>A mutation. Control eyes were obtained from the Lions Eye Bank of Oregon (Portland, OR). The eyes from affected and carrier individuals were obtained at post-mortem examination. The interval between time of death and time of formalin preservation ranged from 5 to12 h. Time in formalin varied from 1 month to 2 years.

For histological analysis, sections were stained with Masson trichrome stain. Immunohistochemistry with anti-neurofilament monoclonal antibodies (Dako, 1:1000) was also performed. For laser capture microdissection (MMI NIKON UV-CUT System, Molecular Machines and Industries) three serial 5-µm thick cut sections were mounted on a polyethylene foil slide and stained with either haematoxylin and eosin or Luxol Fast Blue. Sections were observed under light microscope with a ×40 objective. Selected tissue areas were microdissected by a UV laser and collected on an adhesive cap of nanotubes as previously described (Giordano and d’Amati, 2011). Samples were digested with proteinase K (20 µg/100 µl). Absolute quantification of mitochondrial DNA was performed by the standard curve method using serial known dilutions of a vector in which the mitochondrial DNA region used as template for the amplification (nucleotides 4625–4714) was cloned, as previously detailed (Cossarizza *et al.*, 2003). Preliminary to these experiments, we amplified serial dilutions of DNA obtained from a fixed tissue and we verified that the PCR was quantitative. Nonetheless, as quantity and quality of DNA recovery from formalin-fixed tissues is dramatically influenced by fixation time and type (Gilbert *et al.*, 2007), and as the small amount of DNA obtained from microdissected tissues does not allow one to run an appropriate standard curve within each experiment, to reduce inter-samples variability we report, for each sample, the ratio between the mitochondrial DNA content obtained in two different microdissected areas.

### Statistical analysis

Data were analysed by using SPSS Base 16 software, applying the ANOVA test in conjunction with Tukey’s test. Statistical significance was set at *P < *0.05. Mitochondrial DNA per cell distribution analysis was performed considering the number of individuals falling within classes of 200 mitochondrial DNA copies per cell, graphically expressed as dispersion. Numerical calculations for the normal mixture model, logistic regression and multiple regression analysis were performed with the software R and the package BMA (R: A Language and Environment for Statistical Computing, R Development Core Team, R Foundation for Statistical Computing, Vienna, Austria, 2012, ISBN 3-900051-07-0, http://www.R-project.org/).

## Results

### Analysis of three Leber’s hereditary optic neuropathy pedigrees reveals increased mitochondrial DNA copy number in white blood cells particularly in unaffected mutation carriers

We first investigated mitochondrial DNA copy number in peripheral white blood cells from LHON affected individuals (affected) and unaffected mutation carriers (carriers) from three large LHON pedigrees, two of them previously reported (Carelli *et al.*, 2006), genetically homogeneous for harbouring the m.11778G>A mutation on a haplogroup J background (demographic data of the three families are reported in Table 1 and pedigrees are shown in Supplementary Figs 1–3).

**Figure 1:**
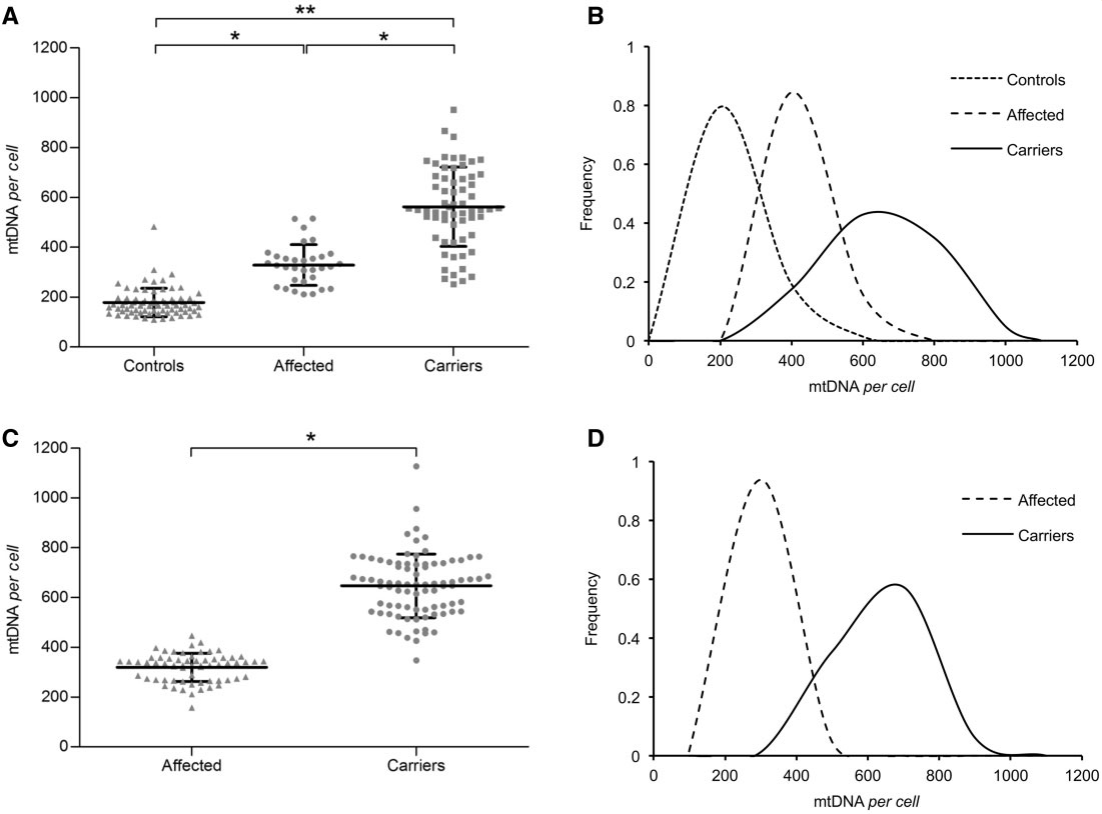
Analysis of mitochondrial DNA (mtDNA) content in three LHON pedigrees and an Italian cohort. (**A**) Scatter-plot of mitochondrial DNA copy number per cell with means ± SD for affected and unaffected mutation carriers from three large LHON pedigrees harbouring the m.11778G>A/*MT-ND4* mutation and for controls. Both affected and carriers showed increased mitochondrial DNA content compared with controls (affected versus controls, *P < *0.0001; carriers versus controls *P < *0.00001). Carriers showed increased mitochondrial DNA content compared with affected individuals (*P < *0.0001). (**B**) Frequency distribution of mitochondrial DNA copy number per cell from the same three families showed that the peak of mitochondrial DNA content shifts towards higher values from controls to affected to carriers. (**C**) Scatter-plot of mitochondrial DNA copies per cell with means ± SD for a large Italian cohort with all the three primary LHON mutations. Unaffected carriers showed increased mitochondrial DNA content compared with affected subjects (*P < *0.0001). (D) Frequency distribution of mitochondrial DNA copy number per cell from the large Italian cohort showed that the peak of mitochondrial DNA content shifts progressively towards higher values from controls to carriers. Experiments were performed in triplicates for all samples. Asterisks indicate statistical significance (*P < 0.0001; **P < 0.00001, ANOVA test).

The cumulative results for all three families, comparing the mitochondrial DNA content per cell of the three groups (affected, carriers and controls) showed that subjects belonging to the mutant maternal lineages (affected and carriers) presented a significantly higher mitochondrial DNA content, as compared with controls (481 ± 18 versus 178 ± 6; *P < *0.0001). Subgrouping the individuals carrying the m.11778G>A mutation into affected and carriers, we found that the mitochondrial DNA content was significantly higher in carriers (Fig. 1A).

By analysing cumulatively the three families, the frequency distribution of the mitochondrial DNA copy number per cell in controls, affected and carriers showed that the peak of mitochondrial DNA content shifts progressively towards higher values from controls to affected to carriers, this last group having the widest variability and peaking at >500 mitochondrial DNA copies (Fig. 1B). This distribution pattern, as well as the statistical significances previously shown amongst groups, is consistent across each of the individual pedigrees (*P < *0.0001) (Supplementary Fig. 4A).

### Analysis of a large Leber’s hereditary optic neuropathy Italian cohort with all three primary mutations confirms that carriers present the highest mitochondrial DNA copy number in white blood cells

To extend the previous findings, we investigated an independent cohort of all available individuals belonging to 39 unrelated Italian LHON pedigrees harbouring one of the three common primary mutations (demographic data of the Italian cohort are reported in Table 2). We assessed the mitochondrial DNA copy number per cell in a total of 64 affected and 68 carriers, finding, as we did in the three large pedigrees described above, a significantly higher mitochondrial DNA content in peripheral white blood cells from carriers compared with affected individuals (Fig. 1C). The frequency distribution of the mitochondrial DNA content for affected and carriers showed carriers peaking at >600 mitochondrial DNA copies (Fig. 1D). Stratifying these data for mutation type, the frequency distribution of the mitochondrial DNA content was similar for the three mutations, with a slightly lower mean for the m.14484T>C carriers (Supplementary Fig. 4B).

### Mitochondrial DNA copies in blood cells discriminate affected and carriers and correlate with subclinical signs of disease in carriers

**Figure 2:**
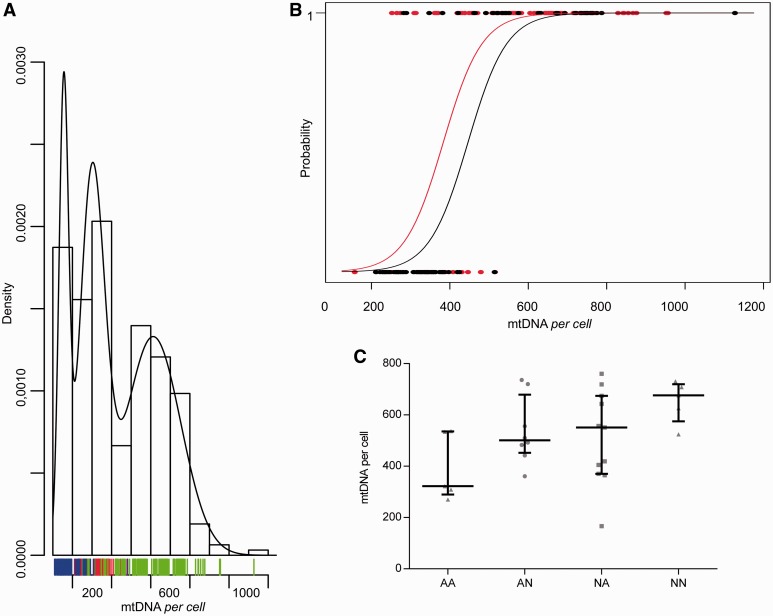
Affected/carrier and gender discrimination by mitochondrial DNA content in blood cells and correlation with subclinical signs of disease in carriers. (**A**) Density of mitochondrial DNA copy number per cell obtained by normal mixture model applied to the overall data set. The model identified three populations that fitted the frequency distribution of controls (blue bars), affected individuals (red bars) and carriers (green bars). (**B**) Logistic regression analysis applied on the same overall data set discriminating affected/carrier status and accounting for gender. Circles in the upper line represent carrier individuals, circles in the bottom line represent affected individuals, red circles are females; black circles are males. The probability of being affected or a carrier is predicted by the blood mitochondrial DNA content with thresholds of >600 and <300 mitochondrial DNA copies per cell, respectively. Females (red line) presented a shifted probability curve as compared with males (black line). (**C**) Scatter-plot of mitochondrial DNA copies per cell with median and interquartile range for a subset of unaffected carriers from the large Family 1, classified using the fundus and the contrast sensitivity evaluation as normal or abnormal (AA = abnormal fundus and abnormal contrast; AN = abnormal fundus, normal contrast; NA = normal fundus, abnormal contrast; NN = normal fundus and normal contrast) (*P* = 0.05 for fundus effect; *P < *0.02 for contrast effect, two-way ANOVA).

After combining all data from the two previous data sets we applied a normal mixture model fitted to the mitochondrial DNA content observations. The model identified three populations with estimated means of 155 ± 24, 301 ± 63 and 612 ± 141 mitochondrial DNA copies per cell (Fig. 2A). These populations consistently fitted the frequency distribution of controls, affected and carriers, with a putative threshold of ∼500 mitochondrial DNA copies discriminating affected from carriers. Logistic regression analysis applied on the same overall data set, stratified by gender, identified a similar mitochondrial DNA copy number per cell threshold. The probability to be a carrier is 100% at >600 mitochondrial DNA copies, whereas <300 the probability of being a carrier is almost reduced to zero (Fig. 2B). Females presented a shifted probability curve indicating that for a certain amount of mitochondrial DNA content the probability to be a carrier is significantly higher compared with males (*P < *0.01), consistent with the known lower risk to be affected in females (Fig. 2B).

It is noteworthy that five carriers from the previously reported large Family 1 (SOA-BR) converted to be clinically affected in the last 10 years, the time elapsing from the blood sampling used in the current investigation (Sadun *et al.*, 2003, 2006). Of these, the four males had <400 mitochondrial DNA copies (277, 289, 359 and 381, respectively), in accordance with the possible threshold of ∼600 mitochondrial DNA copies, above which carriers are at virtually no risk to become affected. The only female, who converted at 46 years of age, had 621 copies of mitochondrial DNA. The blood sample assessed was drawn ∼9 years before she underwent menopause and shortly after she became affected.

For most of the carriers from the large Family 1, we had available a series of ophthalmological examinations, characterizing the subclinical changes that frequently occur in carriers (Sadun *et al.*, 2004; Ventura *et al.*, 2005). Using the fundus examination and the contrast sensitivity evaluation classified as normal or abnormal, we found that mitochondrial DNA copy number was significantly higher in carriers lacking abnormalities in both parameters, as compared with carriers having at least one abnormal evaluation and carriers with both fundus and contrast sensitivity abnormalities, who had the lowest mitochondrial DNA copy number (Fig. 2C). We also assessed by optical coherence tomography the RNFL thickness around the optic nerve head, as well as the optic disc area and the macular RNFL thickness. These are anatomical measures indicative of the number of retinal ganglion cells and correlated axons, which form the RNFL and enter the optic disc. Previous studies documented that a subset of carriers display increased RNFL thickness, probably a result of pathological axonal swelling, in the inferior-temporal quadrants of the optic disc, which is currently considered a subclinical sign of disease (Savini *et al.*, 2005). This increase (pseudoedema) may lead to conversion to visual loss and onset of acute LHON, at which point RNFL thickness decreases as part of the process of optic atrophy (Barboni *et al.*, 2010). However, many carriers maintain lifelong the subclinical increased RNFL thickness without converting. We applied a multiple regression analysis, modelling the mitochondrial DNA content of blood cells as a function of the optical coherence tomography anatomical measures in the carriers from Family 1, and included gender and age as covariates. The best fitting model shows a negative correlation between mitochondrial DNA blood content and the temporal RNFL thickness/nasal-inferior macular thickness (*P < *0.005, Supplementary Fig. 5). Thus, by fixing the other variables, the decreasing mitochondrial DNA content of blood cells correlates with increasing subclinical RNFL thickness of the papillomacular axons originating from the nasal-inferior macula and constituting the temporal quadrant of the optic disc (Supplementary Fig. 5). Another significant correlation in this model positively linked the mitochondrial DNA content with the optic disc area, which has already been shown to be a protective factor for disease conversion and final visual outcome (Ramos *et al.*, 2009).

### Mitochondrial DNA copy number is indicative of global mitochondrial biogenesis

**Figure 3:**
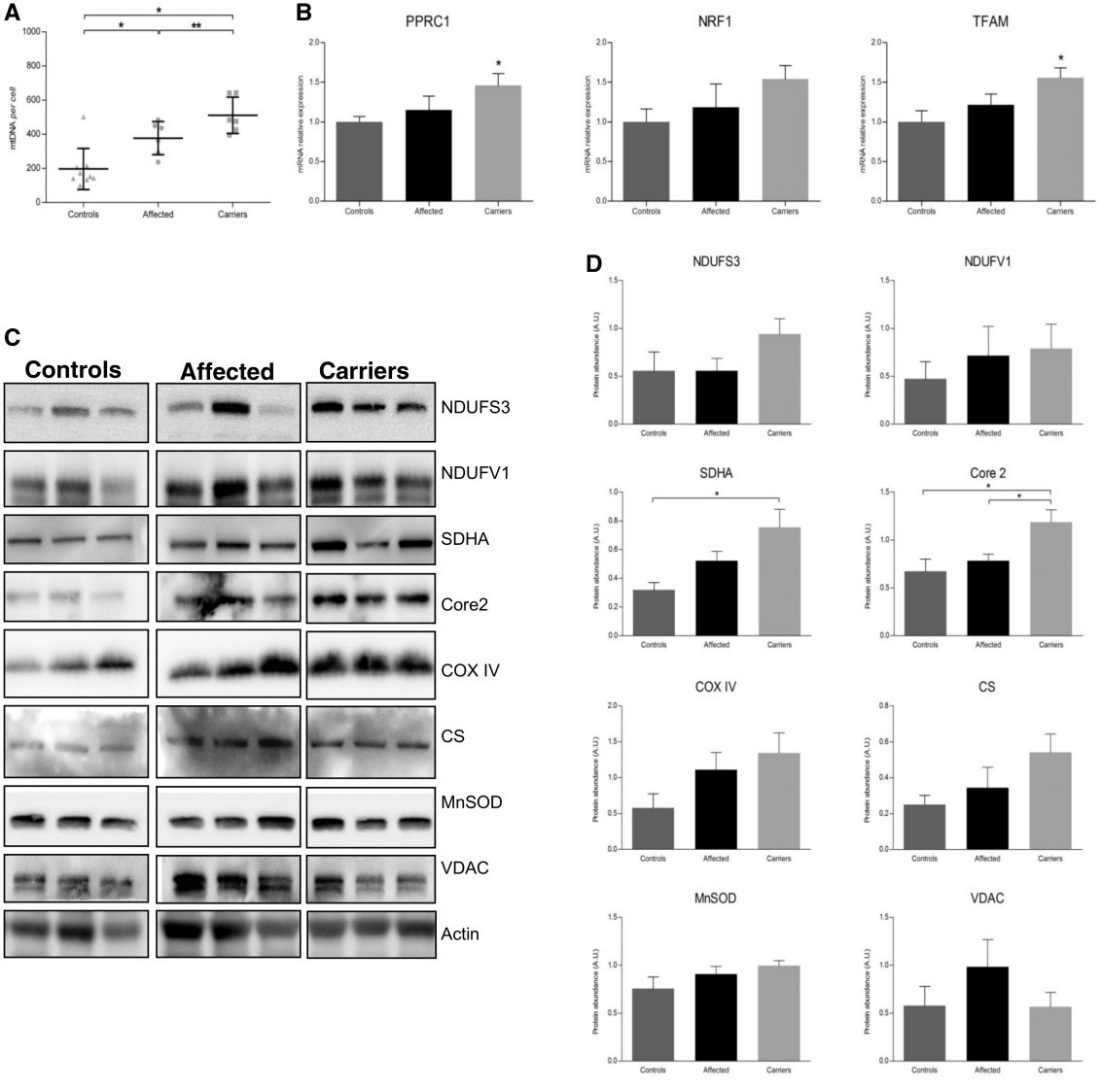
Mitochondrial DNA copy number and global mitochondrial biogenesis in white blood cells. (**A**) Scatter-plot of mitochondrial DNA copy number per cell with mean ± SD. The increase of mitochondrial DNA content was confirmed in both affected individuals and carriers compared with controls and in carriers compared with affected individuals (affected versus controls, *P < *0.01; carriers versus controls, *P < *0.001; carriers versus affected, *P* = 0.05). (**B**) Messenger RNA relative expression (mean ± SEM) of *PPRC1*, *NRF1* and *TFAM*. Significant increase of *TFAM* and *PPRC1* gene expression was found in carriers compared with controls. (**C** and **D**) Mitochondrial proteins expression: representative western blot and densitometry are shown (mean ± SEM). The quantification showed a generalized increase of all proteins content in individuals carrying the LHON mutation compared with controls, higher for carriers, although the statistical significance was not reached. Core2 = Cytochrome b-c1 complex subunit 2; CS = citrate synthetase; MnSOD = manganese superoxide dismutase. Experiments were performed in triplicates for all samples. Asterisks indicate statistical significance: *P < 0.05, **P < 0.01, ANOVA test.

To confirm the activation of global mitochondrial biogenesis in blood cells, we carried out a separate series of experiments from a limited resampled subset of controls (*n = *9), affected (*n = *8) and carriers (*n = *9) belonging to the Italian cohort. For each blood sample, we evaluated the content of mitochondrial DNA and of messenger RNAs coding for key regulators of mitochondrial biogenesis and the amount of proteins belonging to different mitochondrial compartments (matrix, inner and outer mitochondrial membrane). The results are summarized in Fig. 3. The mitochondrial DNA copy number per cell assessment reproduced the previous results, with carriers having the highest mitochondrial DNA content (Fig. 3A). The analysis of gene expression of transcription factors (*NRF1* and *TFAM*) and the *PPRC1* co-activator showed a concordance between mitochondrial DNA content and transcripts level displaying a scale of increasing expression from controls to affected individuals to carriers (Fig. 3B). A significant difference was reached for *PPRC1* and *TFAM*, comparing carriers to controls. Western blots of a panel of representative mitochondrial proteins showed a global increase of all protein content in individuals carrying the LHON mutation compared with controls and in carriers compared with affected individuals (Fig. 3C). However, densitometric analysis failed to show any statistical significance, possibly because of the small data set and the intrinsic interindividual variability (Fig. 3C).

Overall, these results confirmed that blood cells from individuals carrying the LHON mutation have a globally activated mitochondrial biogenesis associated with increased mitochondrial DNA content.

### Increased mitochondrial biogenesis also characterizes the post-mitotic skeletal muscle

**Figure 4:**
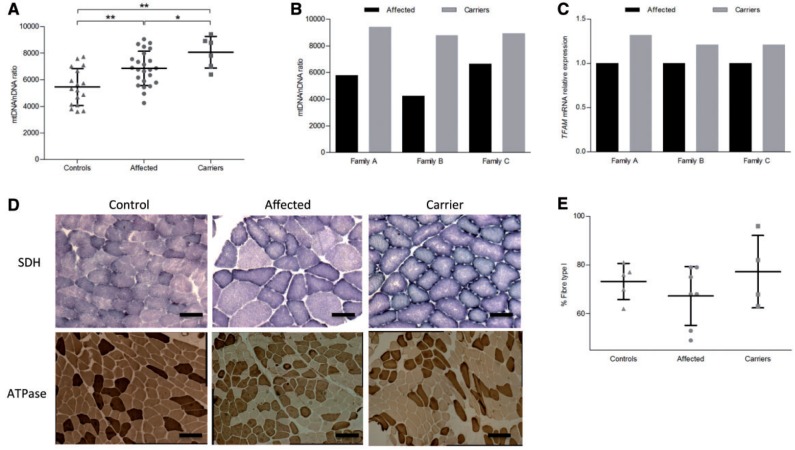
Mitochondrial biogenesis in skeletal muscle from controls, affected individuals and carriers. (**A**) Scatter-plot of mitochondrial DNA/nuclear DNA ratio with mean ± SD for skeletal muscle of controls, affected individuals and carriers belonging to the Italian cohort. A higher mitochondrial DNA content was confirmed in both affected individuals and carriers compared with controls and in carriers compared with affected subjects (affected versus controls, *P < *0.001; carriers versus controls, *P < *0.001; carriers versus affected, *P < *0.05). (**B**) Mitochondrial DNA/nuclear DNA ratio from skeletal muscle in three pairs of discording brothers. All three carriers showed a higher mitochondrial DNA content compared with the affected brother. (**C**) *TFAM* messenger RNA relative expression (mean ± SD) in the skeletal muscle of the three pairs of discording brothers. All three carriers exhibited a higher *TFAM* expression compared with the affected brothers. Experiments were performed in triplicate for all samples. (**D**) Succinic dehydrogenase (SDH) and ATPase pH 9.4 staining in skeletal muscle from controls, affected and carriers. Both affected and carriers presented an increased intensity of subsarcolemmal succinic dehydrogenase staining as compared with controls, especially evident in carriers. Muscle from the affected individual showed variability in fibre size (succinic dehydrogenase, Scale bar = 100 μm; ATPase pH 9.4, Scale bar = 200 μm). (**E**) Scatter-plot of frequency of type I fibres (mean ± SD) in skeletal muscle from controls (*n = *5), affected individuals (*n = *7) and carriers (*n = *4). Asterisks indicate statistical significance: *P < 0.05, **P < 0.01, ANOVA test.

We evaluated the mitochondrial DNA content (expressed as mitochondrial DNA/nuclear DNA ratio) in skeletal muscle biopsies from 17 controls, 24 affected and six carrier individuals harbouring one of the three common primary LHON mutations. In skeletal muscle, as in blood cells, the mitochondrial DNA content was significantly higher in LHON affected individuals and carriers compared with controls, as well as in carriers compared with those affected (Fig. 4A). The difference between affected individuals and carriers was less pronounced compared with the results from blood samples. Interestingly, in considering the three pairs of discordant male siblings available (affected versus carrier brothers), all three male carriers showed a higher mitochondrial DNA content compared with their affected brothers (Fig. 4B). A similar result was obtained by measuring the level of *TFAM* messenger RNA in the same muscle samples (Fig. 4C).

Histoenzymatic staining of the muscle biopsies showed a frequent subsarcolemmal increase of succinic dehydrogenase stain in affected and carriers, indicative of mitochondrial proliferation. These features were more evident in carriers (Fig. 4D). To see if mitochondrial proliferation was associated with a switch in fibre type, we counted type I and II fibres on muscle biopsies from four groups of discordant siblings and from controls (*n = *6). Based on ATPase staining the distribution of muscle fibres was substantially similar in the three groups. Interestingly, two of four carriers showed a higher percentage of type I fibres (82% and 96%, respectively) as compared with affected (range 49–75%) and control subjects (range 62–81%) (Fig. 4D and E).

Taken together, these data confirm that mitochondrial biogenesis also is activated in a post-mitotic tissue with high-energy requirement, exemplified by skeletal muscle.

### Carrier-derived fibroblasts successfully activate mitochondrial biogenesis under forced oxidative phosphorylation conditions

The results obtained on *ex vivo* tissues by comparing affected and carrier individuals prompted us to investigate this issue further *in vitro*, using skin-derived fibroblasts. Most cell studies in LHON used the cybrid cell model (Vergani *et al.*, 1995; Ghelli *et al.*, 2003; Baracca *et al.*, 2005; Floreani *et al.*, 2005; Giordano *et al.*, 2011), where only the mitochondrial DNA from the patient was transferred to a cell line with a constant nuclear background, thus eliminating the effects of the nuclear complement of genes. Hence, we chose to investigate fibroblasts because the hypothetical modifying genes in the nuclear genome might underlie differences in mitochondrial biogenesis measurable by comparing affected and carrier individuals (Guan *et al.*, 1996, 2001). To highlight these differences, we studied fibroblasts grown in medium containing either glucose or galactose as a carbon source. This strategy was previously employed to enhance observable differences in oxidative phosphorylation efficiency, forcing cells in galactose medium to rely on mitochondrial respiration for ATP synthesis (Robinson *et al.*, 1992).

**Figure 5:**
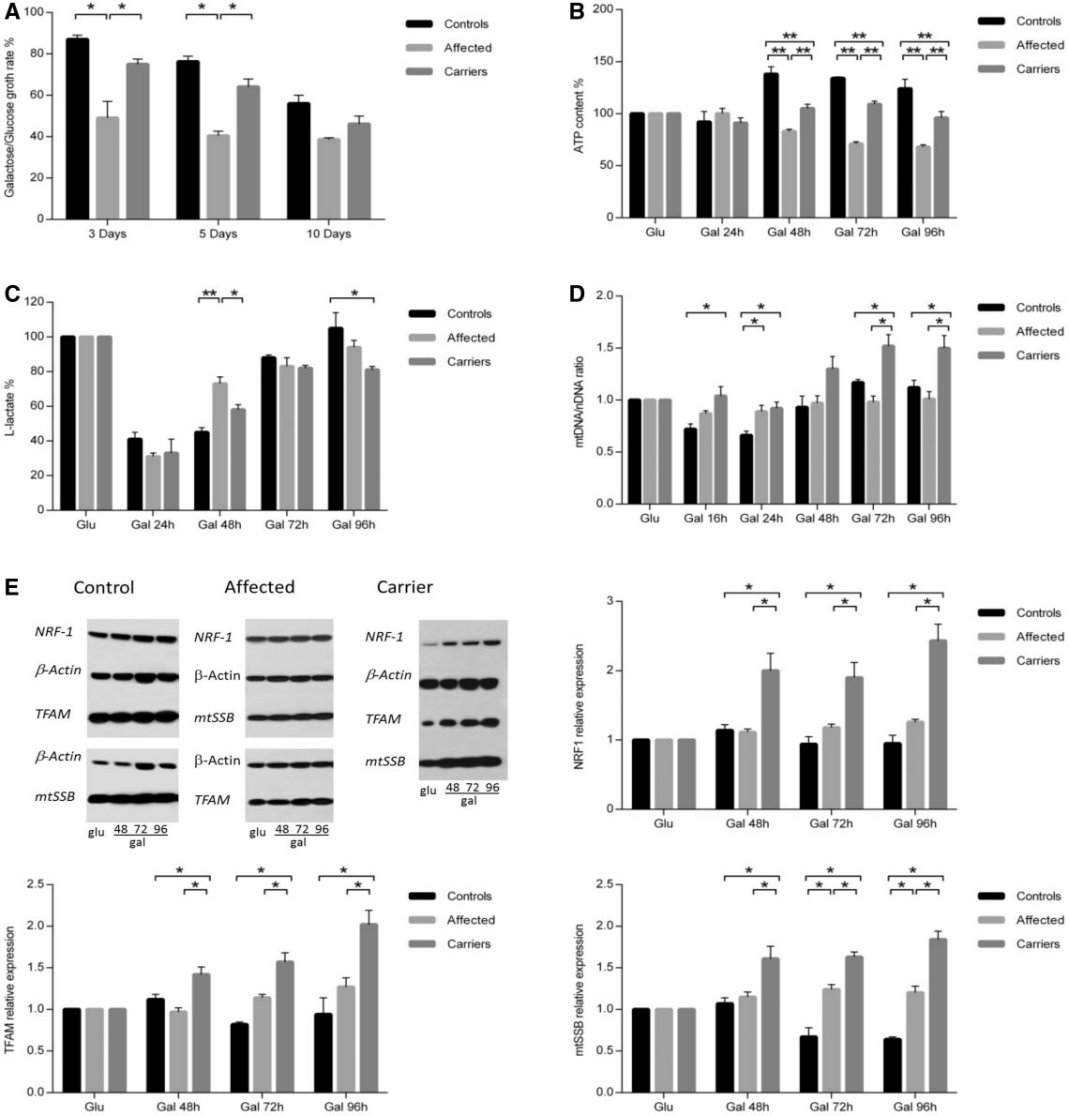
Mitochondrial function and biogenesis in fibroblasts grown in galactose medium. (**A**) Growth rate of controls, affected and carrier-derived fibroblasts reported as the ratio between cell number counted in galactose (Gal) and in glucose (Glu) medium at 3, 5 and 10 days. Carriers significantly differed from affected individuals, which had severely impaired growth, by showing a growth rate closer to controls. (**B**) Intracellular ATP levels during the galactose time course, normalized on ATP level in glucose medium at each time point. ATP content significantly increased in controls and carriers but decreased in affected individuals. (**C**) Level of l-lactate during the galactose time course normalized to the level in glucose at each time point. In all groups l-lactate decreased immediately after switching to galactose medium. At the following time points it increased with a higher rate in affected at 48 h. At the last time point, l-lactate in controls was significantly higher than carriers. (**D**) Mitochondrial DNA amount in fibroblasts (expressed as mitochondrial DNA/nuclear DNA ratio) normalized on mitochondrial DNA amount in glucose medium at each time point. During growth in galactose, carriers significantly increased mitochondrial DNA content as compared with controls and affected individuals. (**E**) Expression of proteins involved in mitochondrial DNA replication (TFAM, mtSSB and NRF1) in galactose medium, normalized on protein levels in glucose medium at each time point. Representative western blot and densitometry are shown (mean ± SEM). Proteins increased in carriers during growth in galactose medium, but remained unchanged in controls and affected individuals. Experiments were performed in quadruplicate for all samples. Asterisks indicate statistical significance: *P < 0.05; **P < 0.01, ANOVA test.

Three control-derived fibroblast cell lines were compared with five affected-derived and four carrier-derived cell lines. The growth rate in galactose medium normalized on growth in glucose at the same time points showed that fibroblasts from controls and carriers grew at a similar rate, whereas a significantly slower rate characterized fibroblasts from affected individuals (Fig. 5A). Moreover, control cell lines activated their ATP synthesis, increasing the ATP levels over the time course in galactose (Fig. 5B). Carriers displayed a similar behaviour, even if less efficiently than controls, whereas affected clearly decreased their ATP content while incubated in galactose (Fig. 5B). The cellular lactate production showed a sharp decrease after switching to galactose medium in all groups, with a progressive increase at the following time points (Fig. 5C). This increase appeared earlier in affected (after 48 h), with a significant difference compared with carriers and controls. Overall, these three experiments underscored a clear difference in oxidative phosphorylation efficiency between affected individuals and carriers. This difference was mirrored by the change in mitochondrial DNA copy number per cell over the time course in galactose medium (Fig. 5D). In fact, the mitochondrial DNA content in fibroblasts from controls and affected individuals had a similar behaviour, failing to show significant changes of mitochondrial DNA copy number. In contrast, fibroblasts from carriers increased their mitochondrial DNA content ∼1.5-fold when incubated in galactose. These results were paralleled by the results of western blot analysis of NRF1, TFAM and mitochondrial single stranded DNA-binding protein (mtSSB), whose levels increased during the time course in galactose medium only in carriers, compatibly with the activation of mitochondrial biogenesis (Fig. 5E).

**Figure 6:**
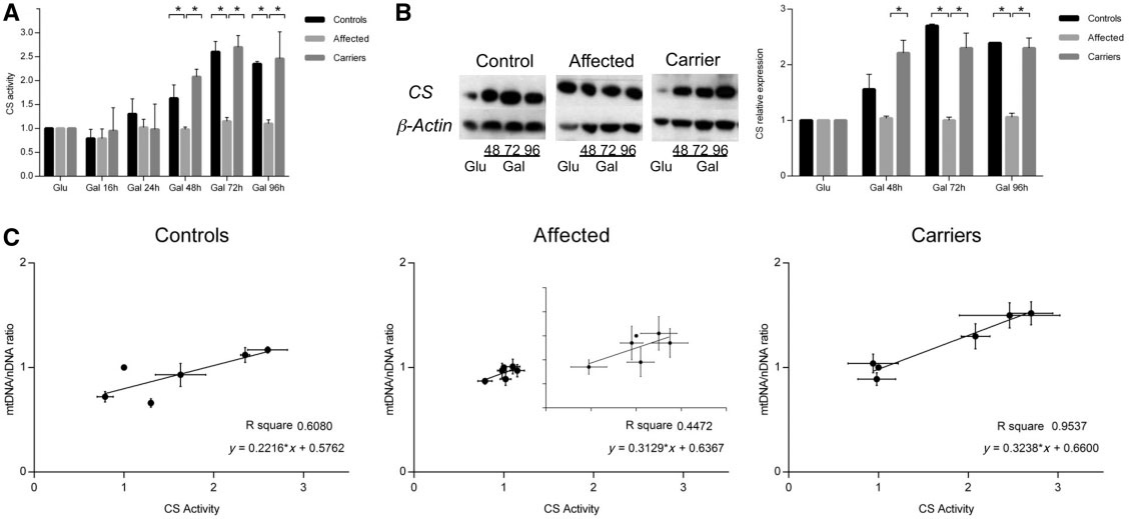
Mitochondrial mass and mitochondrial DNA content correlation in fibroblasts grown in galactose (Gal) medium. (**A**) Citrate synthetase activity (mean ± SEM) measured during the time course in galactose medium normalized on the citrate synthetase activity measured in glucose medium at each time point. Citrate synthetase activity increased after 48-, 72- and 96-h incubation in galactose in controls and carriers, whereas no significant change was observed in the affected group. (**B**) Citrate synthetase protein expression in galactose medium normalized on glucose medium for each time point. Citrate synthetase protein increased after 48-, 72- and 96-h incubation in galactose medium in controls and carriers, whereas it did not change in the affected group. Representative western blot and densitometry are shown (mean ± SEM). (**C**) Correlation between citrate synthetase activity and mitochondrial DNA content during the time course in galactose medium. Only carriers displayed a significant correlation between the increase of mitochondrial mass and mitochondrial DNA amount during the growth in galactose medium. Experiments were performed in triplicate for all samples. Asterisks indicate statistical significance: *P < 0.05; **P < 0.01, ANOVA test.

To further document the increase of mitochondrial biogenesis as a general process not limited to mitochondrial DNA cellular content, we determined the citrate synthase protein level and activity. Control subjects and carriers increased both citrate synthetase activity and protein content in galactose, whereas affected individuals did not show any difference (Fig. 6A and B). In fibroblasts from control and affected subjects, the citrate synthetase activity did not correlate with mitochondrial DNA content (Fig. 6C). Conversely, the increase of citrate synthetase activity and mitochondrial DNA copy number per cell in galactose medium were correlated in carrier-derived cells (Fig. 6C), suggesting a global activation of mitochondrial biogenesis (Fig. 6C).

### Fibroblasts from carriers are the most effective in repopulating mitochondrial DNA after depletion by ethidium bromide

**Figure 7:**
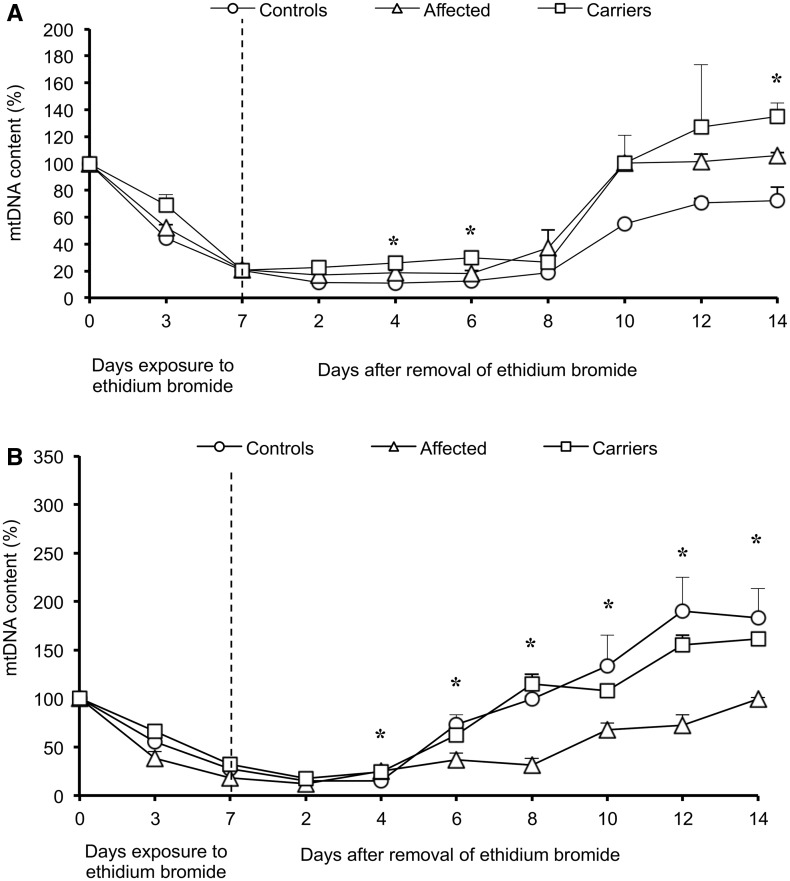
Mitochondrial DNA repopulation after depletion by ethidium bromide in fibroblasts. Mitochondrial DNA depletion and repopulation in glucose (**A**) and in galactose medium (**B**). Mitochondrial DNA content (mean ± SEM) was normalized on Day 0 before ethidium bromide treatment. In glucose medium, after mitochondrial DNA depletion, repopulation started at Day 8. At Day 12, mitochondrial DNA amount increase was most efficient in carriers, followed by affected individuals and controls. In galactose medium, controls had the most efficient repopulation rate, followed by carriers and only one cell line from an affected individual, which had the slowest repopulation rate. Experiments were performed in triplicate for all samples. Asterisks indicate statistical significance: *P < 0.05, ANOVA test.

Recent reports showed that mitochondrial DNA repopulation rate, in cells previously depleted of mitochondrial DNA by ethidium bromide exposure, may vary depending on the efficiency of mitochondrial DNA maintenance machinery (Stewart *et al.*, 2011). We adopted the same experimental strategy to further validate the activation of mitochondrial DNA replication specifically in fibroblasts from carriers. After ethidium bromide withdrawal and growth in glucose medium, cells remained quiescent until Day 8 and then started to increase their mitochondrial DNA content, demonstrating a clear divergence at the 12th day with cells from carriers being the most efficient, followed by those from affected and then control subjects (Fig. 7A). Thus, the presence of the LHON mutation signals a compensatory activation of mitochondrial DNA replication, which is most efficient in carriers with the permissive glucose medium. The faster repopulation in carriers over controls probably depends on the activation of the compensatory response as a result of the LHON mutation. Conversely, when cells were grown in galactose medium after removal of ethidium bromide, control cells showed the most efficient repopulation rate, followed by those from carriers (Fig. 7B). Cells from affected individuals presented a slow repopulation rate until Day 10, at which point three of four lines were lost as a result of massive cell death. The only surviving line continued to show mitochondrial DNA repopulation, albeit at a very slow rate. Thus, in the restrictive galactose medium, mutant cells do perform worst compared with controls.

These results consolidate the evidence for a clear difference between unaffected mutation carriers and affected individuals in activating mitochondrial DNA replication.

### Analysis of human ocular tissues shows that neurodegeneration in Leber’s hereditary optic neuropathy follows the gradient of mitochondrial DNA content and compensatory changes characterize a carrier

To verify our previous findings in the LHON target tissue, we evaluated mitochondrial DNA amount in formalin-fixed retinal and optic nerve post-mortem specimens from six control subjects, one affected and one carrier individual from well-characterized cases belonging to Family 1 (Sadun *et al.*, 2003, 2004; Ventura *et al.*, 2005; Carelli *et al.*, 2006; Sadun *et al.*, 2006; Ramos *et al.*, 2009).

**Figure 8:**
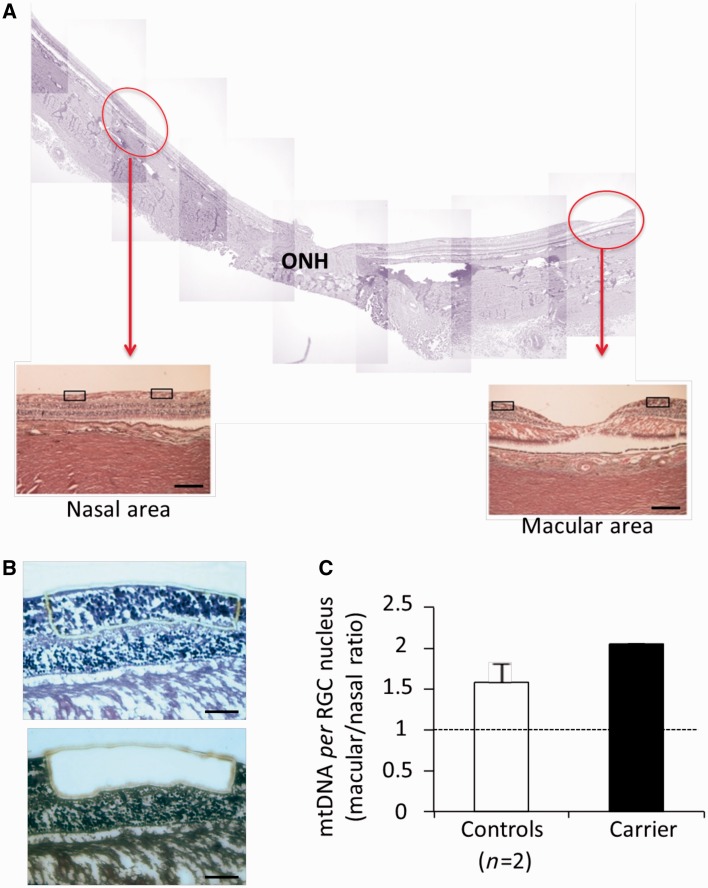
Mitochondrial DNA content in retinal ganglion cells and nerve fibres from the macular and nasal retina. (**A**) Representative montage of horizontal sections through the optic nerve head (ONH) and the macula. Red circles indicate the macular and a nasal area, at equal distance from the optic nerve head, where retinal ganglion cells and the corresponding axons of the RNFL were microdissected by laser capturing for mitochondrial DNA copy number evaluation in two controls and one carrier (haematoxylin and eosin, scale bar = 200 μm). (**B**) Representative image of a retina before and after microdissection of retinal ganglion cells (RGC) and RNFL (haematoxylin and eosin, scale bar = 100μm). (**C**) The macular/nasal mitochondrial DNA ratio indicates that the mitochondrial DNA content (expressed as mitochondrial DNA copy per retinal ganglion cell nucleus) from the macular region is higher as compared with the nasal region. This ratio was even higher in the carrier. Data are mean ± SD from two experiments.

The retinal ganglion cells, selectively affected in LHON, have large soma located in the inner retina, which give origin to unmyelinated axons constituting the RNFL. Each axon then becomes myelinated after turning posteriorly into the optic nerve head and crossing the lamina cribrosa. These myelinated axons form the optic nerve. We first studied, by laser capture, sagittal retinal sections from two controls and the one available carrier, using horizontal sections that went through the optic nerve head and the macula. The retinal ganglion cells and RNFL complex, dissected from the macular region and an equidistant area nasal to the optic nerve head, were compared (Fig. 8A and B). The macular/nasal ratio showed that the mitochondrial DNA content (expressed as mitochondrial DNA copy per retinal ganglion cell nucleus) from the macular region was higher than that from the corresponding nasal region, both in controls and the carrier individuals. The carrier showed the highest ratio (Fig. 8C). These results suggest that the retinal ganglion cells of the macula, which give rise to the axons entering the temporal sector of the optic nerve (i.e. papillomacular bundle), the first target of neurodegeneration in LHON (Sadun *et al.*, 2000), are the most energy-dependent. In addition, the observation that the carrier displays the highest macular/nasal mitochondrial DNA ratio may reflect a possible compensation in macular retinal ganglion cells.

**Figure 9:**
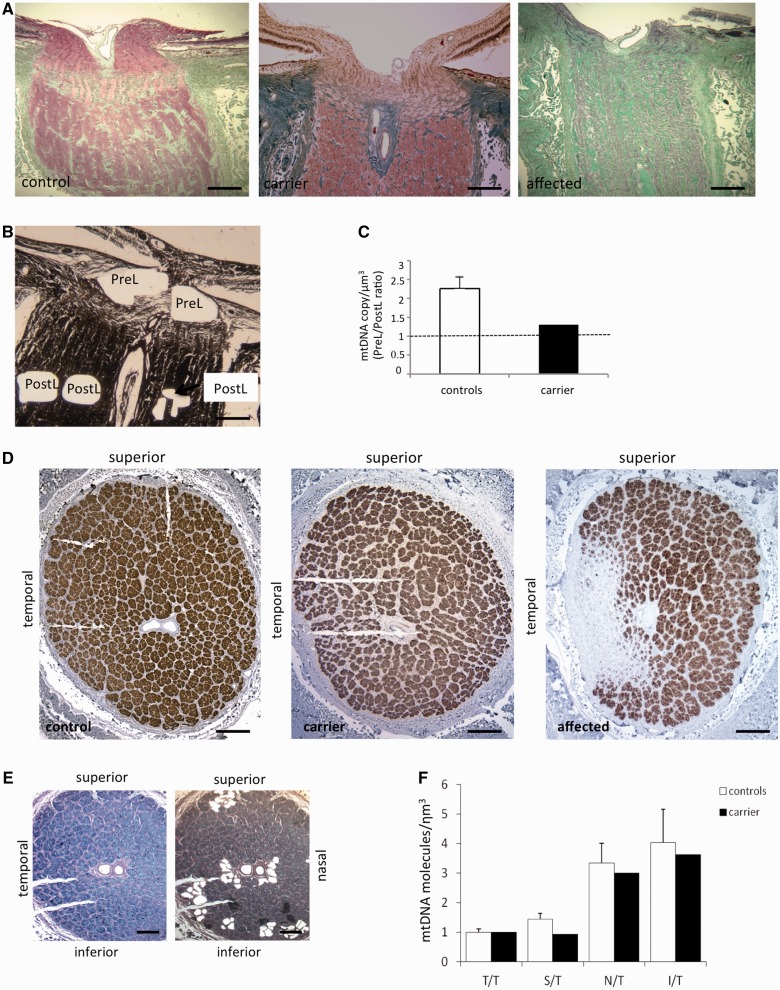
Mitochondrial DNA content in pre/post-laminar regions of sagittal optic nerve head and post-laminar optic nerve cross-sections. (**A**) Sagittal sections through the optic nerve of a control, a carrier and an affected individual. The control and carrier present similar features, whereas the affected subject showed markedly atrophic nerve bundles and a thinned lamina cribrosa (Masson trichrome stain, scale bar = 200 μm). (**B**) Representative sagittal section through the optic nerve head after microdissection of prelaminar (preL) and post-laminar (postL) areas (haematoxylin and eosin, scale bar = 200 μm). (**C**) The pre-/post-laminar mitochondrial DNA ratio indicates a higher mitochondrial DNA density (expressed as mitochondrial DNA molecules per µm^3^) in the pre-laminar region in controls (*n = *6). The carrier had a lower pre-/post-laminar mitochondrial DNA ratio compared with controls. Data are mean ± SD from three experiments.(**D**) Post-laminar optic nerve cross-sections of a control, a carrier and an affected individual. The control and carrier displayed organized bundles of axons. The affected subject showed extensive gliosis of the temporal region and a relative sparing of axons in the other quadrants (anti-neurofilament antibody, scale bar = 500 μm). (**E**) Representative post-laminar optic nerve-cross section before and after microdissection of bundles of axons in the temporal (T, double nick) inferior (I), nasal (N) and superior (S, single nick) quadrants (Luxol Fast Blue, scale bar = 500 μm). (**F**) The superior/temporal, inferior/temporal and nasal/temporal mitochondrial DNA ratio indicate a lower mitochondrial DNA density in the temporal area, both in controls (*n = *6) and the carrier. In the carrier these ratios were slightly lower compared with controls, suggesting a compensatory mitochondrial DNA increase in the temporal quadrant. Data are mean ± SD from three experiments.

Myelination of retinal ganglion cell axons, as they exit the lamina cribrosa, is associated with a marked reduction in the number of mitochondria and activity of respiratory chain enzymes (Andrews *et al.*, 1999; Barron *et al.*, 2004; Carelli *et al.*, 2004; Pan *et al.*, 2012). Thus, we dissected portions of the optic nerve head anterior to the lamina cribrosa and portions of post-laminar myelinated optic nerve using sagittal sections of optic nerves from four controls and the carrier, at similar planes and showing similar features. We did not evaluate the affected individual, because of extensive gliosis (Fig. 9A and B). As expected, evaluation of mitochondrial DNA density (expressed as mitochondrial DNA molecule per µm^3^) showed that, both in controls and the carrier, the prelaminar portion had more mitochondrial DNA copies than the post-laminar region (up to 2.5-fold). Interestingly, the pre/post-laminar ratio was lower in carrier. This might be because of an increased mitochondrial DNA copy number in the carrier’s post-laminar myelinated axons (Fig. 9C).

Finally, we microdissected portions from optic nerve cross-sections in the post-laminar region. Control optic nerves displayed a well-organized cross-sectional profile with axons grouped in ∼1000 fascicles (Carelli *et al.*, 2004). The carrier also showed an essentially conserved number of fascicles and axons. In LHON the neurodegenerative process starts from the temporal sector, which is composed mostly of the small axons from the papillomacular bundle; further degeneration extends into the central region, sparing some of the far periphery of the superior, nasal and inferior quadrants, as shown by the cross-sectional profile of the affected subject here examined and in our previous studies (Fig. 9D) (Sadun *et al.*, 2000; Carelli *et al.*, 2004; La Morgia *et al.*, 2010; Pan *et al.*, 2012). Accordingly, we microdissected samples from the temporal quadrant, as well as from the inferior, nasal and superior far periphery of the optic nerve (Fig. 9E). The results from 12 control optic nerves (six individuals) showed that temporal area had the lowest mitochondrial DNA density (mitochondrial DNA molecules per µm^3^), whereas the nasal and the inferior far periphery of the optic nerve had the highest (Fig. 9F). This pattern is in agreement with our previous observation that the average axonal diameter progressively increases from temporal to nasal regions of the optic nerve, the same gradient followed by the neurodegenerative process (Pan *et al.*, 2012). With the same approach, we also evaluated the carrier individual, who showed a slight decrease of the inferior/temporal, superior/temporal and nasal/temporal ratios compared with controls (Fig. 9F), indicating a compensatory increase of mitochondrial DNA density in the temporal sector (papillomacular bundle), relative to the other sectors of the same nerve. Analysis of the LHON-affected individual revealed the virtual absence of mitochondrial DNA in the temporal sector where the axonal population was ablated, and a decreased mitochondrial DNA level in the nasal and inferior periphery, matching axonal depletion of these areas (data not shown).

### Mitochondrial genetic variability is not a modifying factor of mitochondrial DNA copy number

Multiple reports indicate that specific mitochondrial DNA haplogroup-specific polymorphic changes might influence the abundance of mitochondrial genomes in cells by modulating respiratory chain coupling and reactive oxygen species production (Moreno-Loshuertos *et al.*, 2006; Curran *et al.*, 2007; Suissa *et al.*, 2009; Liou *et al.*, 2010; Gómez-Durán *et al.*, 2012). In particular, haplogroup J and the m.16189T>C/*MT-DLOOP* variant that generates a heteroplasmic uninterrupted polycytosines (poly-C) tract of variable length (Bendall and Sykes, 1995), have been proposed to affect the mitochondrial DNA transcription and replication rates, eventually reflecting on the mitochondrial DNA content (Suissa *et al.*, 2009; Liou *et al.*, 2010).

Thus, we analysed the distribution of mitochondrial DNA content of affected individuals and carriers from the Italian cohort stratified by haplogroup affiliation and presence of the m.16189T>C variant. We failed to observe any significant haplogroup-driven difference, or trend, in mitochondrial DNA copy number (Supplementary Fig. 6). Moreover, none of the analysed samples presented the m.16189T>C polymorphism, indicating that the mitochondrial DNA content variability in our cohort was not determined by this nucleotide change (data not shown).

### Searching for nuclear modifiers: analysis of five candidate genes

These results strongly support a role for mitochondrial biogenesis in modulating LHON penetrance. Thus, we sought to investigate candidate modifiers in some of the genes regulating mitochondrial DNA replication and biogenesis. We selected SNPs already reported in the literature as associated with diseases or having a functional activity. We screened nine SNPs in the coding region of *PPARGC1A* (PGC-1α), *PPARGC1B* (PGC-1β), *TP53* (p53), *TFAM* and *PARL* (Supplementary Table 1). PGC-1α and PGC-1β are transcriptional co-activators involved upstream of NRF1, and TFAM, regarded as master regulators of mitochondrial biogenesis (Scarpulla, 2008). p53 is a tumour suppressor protein mainly involved in the DNA repair system, also implicated in the regulation of mitochondrial DNA replication by interacting with POLG (Achanta *et al.*, 2005). TFAM, the main mitochondrial DNA transcription factor, is the most abundant nucleoid protein and regulates mitochondrial DNA replication (Campbell *et al.*, 2012). Finally, PARL is a mitochondrial protease, which cleaves, among others, OPA1 and PINK1, genes responsible for dominant optic atrophy and familial Parkinson’s disease (Martinelli and Rugarli, 2010). A recent report implicated a specific *PARL* SNP in regulating mitochondrial DNA copy number in a control population (Curran *et al.*, 2010), and linkage analysis in Asian LHON pedigrees found a significant association with other *PARL* SNPs (Phasukkijwatana *et al.*, 2010). Thus, we screened these genetic variants in the large Family 1, in the cohort of 39 Italian families and in a further, independently collected, LHON cohort from Northern Europe. The affected and carrier individuals were compared, considering genotypes (homo/heterozygous) and allelic frequencies. We also searched for a correlation between genotypes and mitochondrial DNA content and a multiple logistic regression of all SNPs was performed.

None of the genotypes, or the allelic frequencies showed a statistical difference between the two groups of affected and carriers (Supplementary Table 1). Interestingly, we found a significant correlation between two SNPs in the promoter of *PARL* gene (rs3792588 and rs3792589) and the mitochondrial DNA cellular content of affected individuals belonging to the large Family 1 (Supplementary Fig. 7), but not in the affected individuals of the Italian cohort (data not shown).

## Discussion

We have demonstrated, using different approaches, that mitochondrial DNA content and mitochondrial biogenesis are associated with incomplete penetrance in homoplasmic LHON families. First, we showed that high mitochondrial DNA content and increased mitochondrial biogenesis, both in high turnover tissue such as peripheral white blood cells and in post-mitotic tissue such as skeletal muscle, are significantly associated with unaffected LHON mutation carriers. In blood cells, a threshold of ∼500 mitochondrial DNA copies discriminates the probability of an individual carrying the LHON mutation to remain unaffected or undergo clinical conversion to optic atrophy. Furthermore, in carriers the mitochondrial DNA blood content was inversely correlated with the occurrence of subclinical pathological features such as fundus abnormalities and contrast/sensitivity losses, and with structural abnormalities as measurements by optical coherence tomography. We further demonstrated that fibroblasts derived from LHON carriers, grown under the metabolic pressure of galactose medium or after ethidium bromide-driven mitochondrial DNA depletion, activate mitochondrial biogenesis and mitochondrial DNA replication more efficiently compared with those from affected individuals. Finally, our results on post-mortem specimens indicate that the pattern of neurodegeneration in the optic nerve is inversely related to the axonal mitochondrial DNA density, which in turn matches the axonal diameter (Sadun *et al.*, 2000; Pan *et al.*, 2012). Thus, the small fibres that constitute the papillomacular bundle, the first target in LHON, by virtue of their high surface/volume ratio are limited in their capacity for compensation and this explains their high vulnerability (Sadun *et al.*, 2000; Pan *et al.*, 2012). In addition, in a single carrier, concordant results from the optic nerve and retinal sections suggest the occurrence of a compensatory increase in mitochondrial DNA amount in the most vulnerable macular retinal ganglion cells and their axons that form the papillomacular bundle, which runs into the temporal optic nerve head*.* These results lend support to the hypothesis that carrier individuals are more likely to achieve, by genetic variation, a highly efficient mitochondrial biogenesis. This genetic modifying background may further interact with environmental (Sadun *et al.*, 2003; Carelli *et al.*, 2004) and hormonal factors (Giordano *et al.*, 2011), ultimately determining the outcome of an LHON carrier in becoming affected. We tested the possibility that genetic variants in mitochondrial DNA itself or in the nuclear-encoded molecular machinery controlling mitochondrial DNA replication and mitochondrial biogenesis, may play a modifying role leading to incomplete penetrance in LHON; we evaluated mitochondrial DNA haplogroups and screened a few candidate SNPs of interest in nuclear genes, without finding any significant association in this pilot study. Overall, we have established a solid correlation between incomplete penetrance in LHON and the efficiency of mitochondrial biogenesis. This observation may broadly apply to other mitochondrial DNA mutations with a weak pathogenic potential.

LHON pathogenic mutations invariably affect complex I with different biochemical phenotypic consequences (Carelli *et al.*, 2004; Newman, 2005). The m.3460G>A mutation consistently decreases the complex I specific activity by affecting the ND1 subunit and the quinone binding site, whereas the m.11778G>A and m.14484T>C mutations, affecting the ND4 and ND6 subunits, respectively, do not decrease complex I activity, but most probably interfere with proton translocation and complex I-dependent energy conservation (Carelli *et al.*, 2004; Gonzalez-Halphen *et al.*, 2011). All three LHON primary mutations ultimately decrease complex I-driven ATP synthesis rate, and in addition, chronically increase reactive oxygen species production and predispose cells to apoptosis (Vergani *et al.*, 1995; Ghelli *et al.*, 2003; Baracca *et al.*, 2005; Floreani *et al.*, 2005). Different views have emphasized the bioenergetic defect as most important or, conversely, hypothesized that the critical problem resided in increased reactive oxygen species production. Recent data, obtained by studying the first faithful mouse model of LHON, added weight to oxidative stress as the critical pathogenic mechanism for retinal ganglion cells neurodegeneration (Lin *et al.*, 2012). However, increased reactive oxygen species production, in addition to damaging cells, may also signal mitochondrial biogenesis and mitochondrial DNA content (Moreno-Loshuertos *et al.*, 2006). This signalling system, originating from dysfunctional mitochondria, may activate an orchestrated response with different efficiencies based on genetic variation in the complex genetic machinery involved in mitochondrial biogenesis and mitochondrial DNA replication (Liu and Butow, 2006). Thus, increased mitochondrial biogenesis may be a compensatory response that, if sufficiently efficient, maintains unaffected the mutation carriers (Korsten *et al.*, 2010), even if we cannot completely exclude other factors differentiating carriers and affected, such as mitophagy rate and mitochondrial turnover.

Given the overall weak pathogenic potential of LHON mutations, we envisage a scenario where increased mitochondrial mass supports the bypassing of complex I, through complex II and downstream complexes III and IV, which are all unaffected by the LHON mutations. This and the parallel increase of antioxidant enzymes such as manganese superoxide dismutase and glutathione peroxidase, as we also documented after oestrogen exposure of LHON cybrids (Giordano *et al.*, 2011), may coordinately balance the complex I dysfunction and maintain unaffected the carriers. If this compensatory mechanism fails, the increased reactive oxygen species production may synergize with deficient bioenergetics, ultimately leading to retinal ganglion cell loss, thus converting mutation carriers to affected status.

Our current results closely resemble those recently reported for an incompletely penetrant mitochondrial DNA mutation affecting the transfer RNA isoleucine gene (Moreno-Loshuertos *et al.*, 2011). Furthermore, seminal cybrid studies by Bentlage and Attardi (1996) highlighted the role played by mitochondrial DNA content in modulating the pathogenic potential of the m.3243A>G/*MT-TL1* mutation associated with mitochondrial encephalopathy, lactic acidosis, stroke-like syndrome (MELAS). Similarly, most homoplasmic cybrids carrying the deafness-associated mitochondrial DNA mutation at position m.7473insC/*MT-TS1* displayed only a mild respiratory defect, with the exception of one clone with a reduced mitochondrial DNA content that expressed severe growth impairment in galactose medium (Toompuu *et al.*, 1999).

Two further observations from our study deserve comment. First, the m.14484C>T/*MT-ND6* mutation had a lower average peak of mitochondrial DNA content in blood cells for carriers. This is compatible with the milder nature of this mutation, prone to low penetrance in females and high rates of spontaneous recovery of vision (Carelli *et al.*, 2004; Newman, 2005). Second, the only female who converted to become affected in Family 1 had a supra-threshold mitochondrial DNA content (621 copies) in her blood cells, but she became affected after her menopause, and 9 years after her blood was sampled. We postulate a post-menopausal decrease in mitochondrial DNA content triggered her conversion to affected, in accordance with the protective role of oestrogens in LHON. In fact, we have recently shown in cybrid cells that oestrogens activate mitochondrial biogenesis and mitochondrial DNA content (Giordano *et al.*, 2011), a result both compatible with these present observations and the low penetrance of LHON in females.

One important implication of these findings is that the assessment of mitochondrial DNA cellular content may, once solidly validated by quantitative studies in different tissues, become a surrogate biomarker with predictive value on metabolic compensation of unaffected mutation carriers; this would be in conjunction with other clinical and metabolic markers. The combination of predictive markers may lead to a scoring system useful in the assessment of prognosis and therapeutic options. Validation of the current results, by dissecting the molecular mechanism underlying the retrograde signalling that regulates mitochondrial biogenesis and the understanding of genetic determinants driving the efficiency of mitochondrial biogenesis, will also be advantageous to exploit this spontaneous compensatory strategy for therapeutic interventions. In fact, several studies are testing the feasibility of this approach, which in the case of LHON may allow unaffected carriers to avoid conversion indefinitely.

Our targeted approach of screening selected variants in candidate genes did not lead to positive results. We failed to confirm the association with the previously reported SNPs in the *PARL* gene, which were attractive candidates considering the proposed role played by this protease in controlling mitochondrial DNA copy number (Curran *et al.*, 2010). Similarly, we failed to observe any stringent association with mitochondrial DNA haplogroups or specific variants (Moreno-Loshuertos *et al.*, 2006; Curran *et al.*, 2007; Suissa *et al.*, 2009; Liou *et al.*, 2010; Gómez-Durán *et al.*, 2012). However, currently available high-throughput strategies of genetic screening, as well as expression studies in relevant tissues, using large cohorts of patients or informative pedigrees, increase the probability of successfully identifying the genetic basis of incomplete penetrance in LHON. It must be considered that a complex interaction of genetic variants with environmental factors, already validated as triggers for LHON, may further complicate their identification.

In conclusion, we provide multiple lines of evidence that cellular mitochondrial DNA content can differentiate the LHON affected individuals from the unaffected mutation carriers. This observation supports a mechanism in which penetrance is modulated by the ability to efficiently activate mitochondrial biogenesis. This is a natural compensatory strategy that cells from carriers successfully use. This mechanism has wide implications for the pathogenesis of LHON, providing indications for hunting the nuclear genetic modifiers and studying their possible interaction with environmental triggers. Ultimately, this compensatory strategy may be exploited for new therapies in LHON carriers to maintain their visual function indefinitely.

## Supplementary Material

Supplementary Data

## Abbreviations

LHON: Leber’s hereditary optic neuropathy
RNFL: retinal nerve fibre layer
SNP: single nucleotide polymorphism
