# Supplementary material for: Efficient mitochondrial biogenesis drives incomplete penetrance in Leber’s hereditary optic neuropathy
Source: Brain. 2013 Dec 24;137(2):335–53. doi: 10.1093/brain/awt343 (PMC3914475; doi:10.1093/brain/awt343)
Supplement: Supplementary Data [file supp_137_2_335__index.html]

Efficient mitochondrial biogenesis drives incomplete penetrance in Leber’s hereditary optic neuropathy — Supplementary Data 

# Efficient mitochondrial biogenesis drives incomplete penetrance in Leber’s hereditary optic neuropathy

## Supplementary Data

files

**Files in this Data Supplement:**

- Supplementary Data - doc file
