# Supplementary material for: Efficient mitochondrial biogenesis drives incomplete penetrance in Leber’s hereditary optic neuropathy
Source: Brain. 2013 Dec 24;137(2):335–53. doi: 10.1093/brain/awt343 (PMC3914475; doi:10.1093/brain/awt343)
Supplement: Supplementary Data [file supp_awt343_brain-2013-01141-File011.doc]

Efficient mitochondrial biogenesis drives incomplete penetrance in

Leber’s hereditary optic neuropathy

**SUPPLEMENTARY MATERIAL**

***Evaluation of mtDNA copy number by quantitative Real Time PCR***

Total DNA was extracted from whole blood, having isolated an enriched white blood cells fraction after red blood cells lysis and pelleting cells at 3000 rpm for 10 minutes (3 times), or muscle biopsies by phenol-chloroform standard procedures. Quantitative Real Time PCR (qRT-PCR) was used to assess mtDNA content, by using a previously described method (Mussini *et al*., 2005). Total DNA from fibroblasts was extracted using the Wizard® Genomic DNA Purification Kit (Promega) following the manufacturer's instructions. mtDNA copy number quantification in fibroblasts was performed by a qRT-PCR method based on TaqMan® probe chemistry, amplifying *MT-ND1* and *ACTB* genes. Sequence of oligonucleotides and PCR conditions are available upon request. The relative quantification of mtDNA was performed according the Pfaffl mathematical model (Pfaffl, 2001).

***Fibroblast cell lines and culture conditions***

Fibroblast primary cultures were established from skin biopsies from five affected, four carriers and three controls. Fibroblast were grown in high glucose DMEM medium (Euro-Clone) supplemented with 10% fetal bovine serum (FBS) (Euro-Clone), 2mM L-glutamine, 100U/mL penicillin and 100U/ml streptomycin (glucose-medium) and maintained at 37°C in an incubator under a humidified 5% CO_2_ atmosphere. For the experiments in galactose, the glucose-DMEM was replaced after 48 hours with fresh glucose-free DMEM medium (Life Technologies) supplemented with 5mM galactose, 5mM Na-pyruvate, and 5% FBS (galactose-medium).

***Cell viability***

Cell proliferation was measured by Trypan Blue dye exclusion assay. 1x10^5^ cells were seeded in dishes and grown in parallel in glucose-medium and galactose-medium for 3, 5 and 10 days. At these time points cells were detached, washed in PBS, resuspended in Trypan Blue solution at 1:1 ratio and counted using a hematocytometer.

***mtDNA repopulation after ethidium bromide treatment***

Cells were grown in glucose-medium supplemented 0.05mg/ml uridine and 50ng/ml ethidium bromide (EtBr) to induce mtDNA depletion. After 7 days, EtBr was removed from the medium and cells were propagated until 14^th^ day in glucose-medium or galactose-medium supplemented with 0.05mg/ml uridine. All cells were kept at 80% confluence with excess fresh medium to ensure exponential growth.

***Measurement of citrate synthase activity, cellular ATP content and L-lactate levels***

CS activity (nmol x min^-1^ x mg cell protein ^-1^) was measured as previously reported (Srere, 1969) and normalized on protein content determined by Bradford assay (Bradford, 1976). ATP levels were determined using the luciferin/luciferase assay in GloMax 20/20 Luminometer (Promega) as described elsewhere (Manfredi *et al*., 2002; Zanna *et al*., 2005).

L-lactate was spectrophotometrically determined at 340 nm monitoring the formation of NADH during its oxidation to pyruvate in the presence of lactate dehydrogenase (LDH). Briefly, aliquots of the supernatant were added to a reaction mixture containing 1mM NAD^+^, 20U heart bovine LDH, 0,2M glycine and 0,15M hydrazine, pH 9.5.

***Gene expression assay***

Total RNA from peripheral blood was extracted with the QIAmp RNA blood (QIAGEN) and reverse transcribed, using Transcriptor First Strand cDNA Synthesis Kit (Roche Diagnostics) following the manufacturer protocol. Total RNA from 20mg of frozen muscle was extracted by using RNeasy Fibrous Tissue (QIAGEN) and reverse-transcribed with enhanced avian HS RT-PCR kit (Sigma-Aldrich), according to the manufacturer instruction. Expression levels of *PPRC1*, *NRF1* and *TFAM* were determined by qRT-PCR using Universal Probe Library (UPL) assays or SYBR Green I chemistry (Roche Diagnostics) and normalized on *TUBB* levels as reference gene. Oligonucleotides sequences and PCR conditions are available upon request.

***Protein extraction and Western blotting***

Cells were lysed in 1X RIPA Buffer (50mM Tris-HCl pH 7.6, 150mM NaCl, 1% NP40, 1% Na-Deoxycholate, 0.1% SDS, 5mM EDTA), with protease inhibitors (5mg/ml pepsatin, 25mg/ml leupeptin; 5mg/ml antipain and 25mg/ml chymostatin) and 1mM PMSF. Equal quantities of proteins (10µg) were separated by electrophoresis in a 4-12% SDS-polyacrylamide gradient gel (CriterionTM XT Precasted Gels, Biorad) and transferred onto a PVDF membrane (Millipore). Membranes were blocked with 5% non-fat dry milk in PBS containing 0.1% Tween20 and incubated at 4°C overnight with anti-human TFAM (gift from Prof Wiesner, University of Koln, 1:2000), anti-human mtSSB (gift from Prof Kang, Kyushu University, 1:3000), anti-human NRF1 (Abnova Corporation, 1:3000), anti-human SOD2 (Stressgene, 1:200000), anti-human CS (Alpha Diagnostic, 1:1000), anti-human SDHA (Mitosciences, 1:10000), anti-human VDAC (Abcam, 1:1000), anti-human COX IV (Mitosciences, 1:1000), anti-human Core 2 (Mitosciences, 1:1000), anti-human NDUFV1 (Sigma-Aldrich, 1:1000) and anti-human b-actin (Sigma-Aldrich, 1:100000). The proper secondary antibodies were goat anti-rabbit or goat anti-mouse (Santa Cruz or Jackson Immunoresearch). The immunoreactivity was detected using the chemiluminescent substrate ECL (Amersham Biosciences) and densitometry was performed using the Quantity One software (BioRad) and by laser densitometry, using the LKB-Pharmacia Ultroscan XL Densitometer.

***Genetic variability screening***

mtDNA profiles were determined by sequencing the entire mtDNA control region for each subject from nucleotide position (np) 16024 to np 576. This was followed by a hierarchical survey of haplogroup and sub-haplogroup diagnostic markers in the coding region (Giordano *et al*., 2011). SNPs of *TP53*, *PARL* and *TFAM* were analyzed in Family 1 and in the Italian cohort through TaqMan® SNP Genotyping Assays (Life Technologies, Milan, Italy) (Supplementary Table 2). The *PPARGC1A* rs8192678 and *PPARGC1B* rs7732671 were screened in the SOA-BR and in the Italian families by PRC amplification followed by restriction fragment length polymorphism (RFLP) analysis. Primer sequences and enzymatic digestion conditions are available upon request.

**Supplementary References**

Bradford MM. A rapid and sensitive method for the quantitation of microgram quantities of

protein utilizing the principle of protein-dye binding. Anal Biochem. 1976; 72:248-54

Giordano C, Montopoli M, Perli E, Orlandi M, Fantin M, Ross-Cisneros FN, et al. Oestrogens

ameliorate mitochondrial dysfunction in Leber's hereditary optic neuropathy. Brain 2011;

134:220 234.

Manfredi G, Yang L, Gajewski CD, Mattiazzi M. Measurements of ATP in mammalian cells.

Methods 2002; 26(4):317-26.

Mussini C, Pinti M, Bugarini R,  [Borghi V](http://www.ncbi.nlm.nih.gov/pubmed?term=Borghi V%5BAuthor%5D&cauthor=true&cauthor_uid=16184032), [Nasi M](http://www.ncbi.nlm.nih.gov/pubmed?term=Nasi M%5BAuthor%5D&cauthor=true&cauthor_uid=16184032), [Nemes E](http://www.ncbi.nlm.nih.gov/pubmed?term=Nemes E%5BAuthor%5D&cauthor=true&cauthor_uid=16184032), et al. Effect of CD4-monitored

treatment interruption on mitochondrial DNA content in HIV-infected patients: a prospective

study. AIDS 2005; 19(15):1627-33.

Pfaffl MW. A new mathematical model for relative quantification in real-time RT-PCR. Nucleic

Acids Res 2001; 29(9):e45.

Srere PA. Citrate synthase. Methods Enzymol 1969; 13:3–11.

.

Zanna C, Ghelli A, Porcelli AM, Martinuzzi A, Carelli V, Rugolo M. Caspase-independent death

of Leber's hereditary optic neuropathy cybrids is driven by energetic failure and mediated by AIF and Endonuclease G. Apoptosis 2005; 10(5):997-1007.

**Supplementary Table 1.** SNPs and genotype frequency results in the three data sets, with relative p value.

| **Gene** | **SNP** |  | **Family 1** | | **Italian Cohort** | | **UK Cohort** | |
| --- | --- | --- | --- | --- | --- | --- | --- | --- |
| **PPARGC1A** | rs8192678  c.1444G>A p.Gly482Ser |  | Affected | Carriers | Affected | Carriers | Affected | Carriers |
|  |  | GG | 32% | 12% | 48% | 38% | 34% | 43% |
|  |  | GA | 52% | 65% | 37% | 48% | 55% | 44% |
|  |  | AA | 16% | 24% | 15% | 14% | 10% | 13% |
|  |  | p value | 0,16 | | 0,4 | | 0,66 | |
| **PPARGC1B** | rs7732671  c.490G>C p.Ala164Pro |  | Affected | Carriers | Affected | Carriers | Affected | Carriers |
|  |  | GG | 86% | 80% | 69% | 77% | 90% | 89% |
|  |  | GC | 14% | 20% | 31% | 23% | 10% | 11% |
|  |  | CC | 0% | 0% | 0% | 0% | 0% | 0% |
|  |  | p value | 0,73 | | 0,61 | | 0,87 | |
| **TP53** | rs1042522  c.215C>G p.Pro72Arg |  | Affected | Carriers | Affected | Carriers | Affected | Carriers |
|  |  | CC | 17% | 31% | 51% | 49% | 7% | 0% |
|  |  | CG | 67% | 59% | 35% | 41% | 28% | 49% |
|  |  | GG | 17% | 9% | 15% | 10% | 65% | 51% |
|  |  | p value | 0,39 | | 0,45 | | 0,51 | |
| **TFAM** | rs1937  c.35G>C p.Ser12Thr |  | Affected | Carriers | Affected | Carriers | Affected | Carriers |
|  |  | GG | 61% | 81% | 80% | 78% | 69% | 65% |
|  |  | CG | 39% | 19% | 18% | 22% | 31% | 31% |
|  |  | CC | 0% | 0% | 2% | 0% | 0% | 4% |
|  |  | p value | 0,17 | | 0,29 | | 0,45 | |
| **PARL** | rs1402000  c.321+112A>G |  | Affected | Carriers | Affected | Carriers | Affected | Carriers |
|  |  | CC | 21% | 31% | 45% | 46% | 48% | 36% |
|  |  | CT | 54% | 38% | 46% | 51% | 40% | 47% |
|  |  | TT | 25% | 31% | 8% | 3% | 12% | 16% |
|  |  | p value | 0,45 | | 0,2 | | 0,22 | |
|  | rs3792588  g.183602831C>G |  | Affected | Carriers | Affected | Carriers | Affected | Carriers |
|  |  | CC | 83% | 88% | 80% | 76% | 2% | 0% |
|  |  | CG | 17% | 13% | 20% | 21% | 22% | 15% |
|  |  | GG | 0% | 0% | 0% | 2% | 76% | 85% |
|  |  | p value | 0,71 | | 0,19 | | 0,18 | |
|  | rs3792589  g.183602825A>G |  | Affected | Carriers | Affected | Carriers | Affected | Carriers |
|  |  | AA | 83% | 88% | 80% | 75% | 89% | 87% |
|  |  | AG | 17% | 13% | 19% | 22% | 9% | 13% |
|  |  | GG | 0% | 0% | 1% | 2% | 2% | 0% |
|  |  | p value | 0,71 | | 0,48 | | 0,92 | |
|  | rs3749446  c.126-157G>A |  | Affected | Carriers | Affected | Carriers | Affected | Carriers |
|  |  | TT | 16% | 25% | 46% | 48% | 47% | 37% |
|  |  | CT | 60% | 41% | 46% | 48% | 41% | 51% |
|  |  | CC | 24% | 34% | 8% | 4% | 12% | 12% |
|  |  | p value | 0,35 | | 0,34 | | 0,49 | |

**Supplementary Figure 1.** Family 1 pedigree


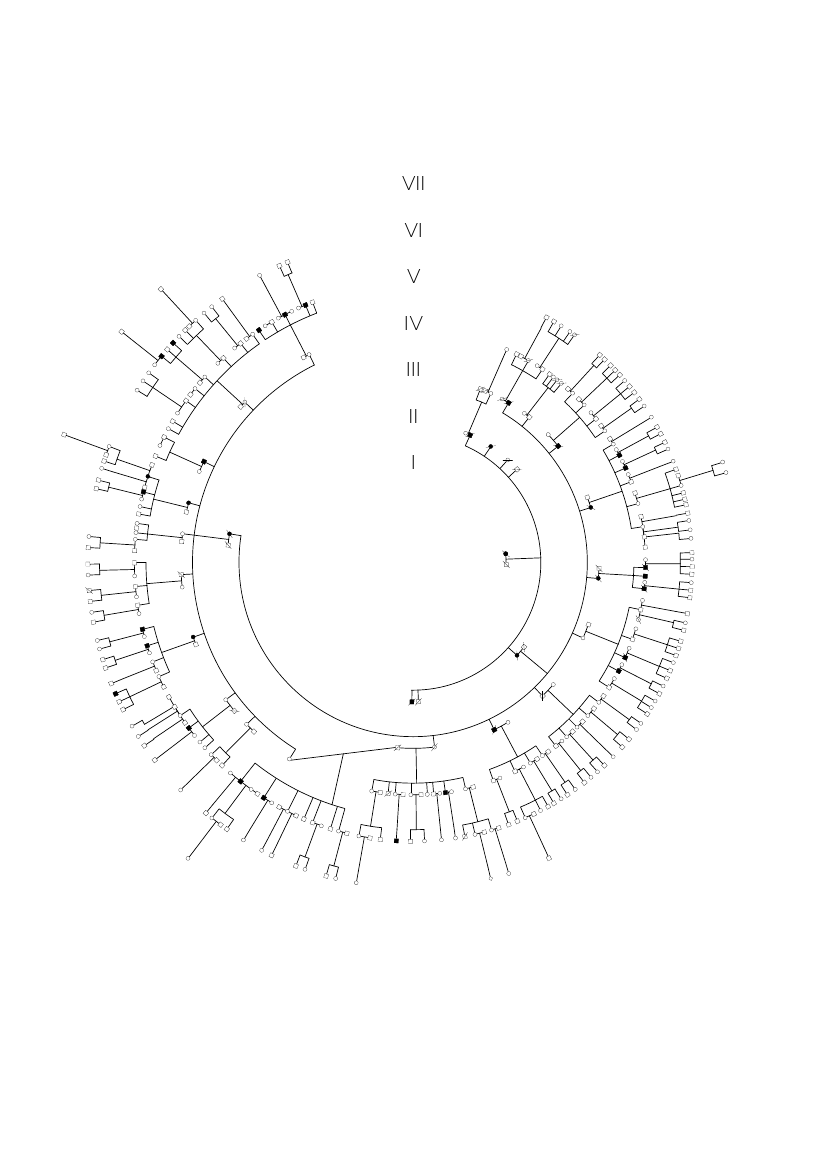


**Supplementary Figure 2.** Family 2 pedigree


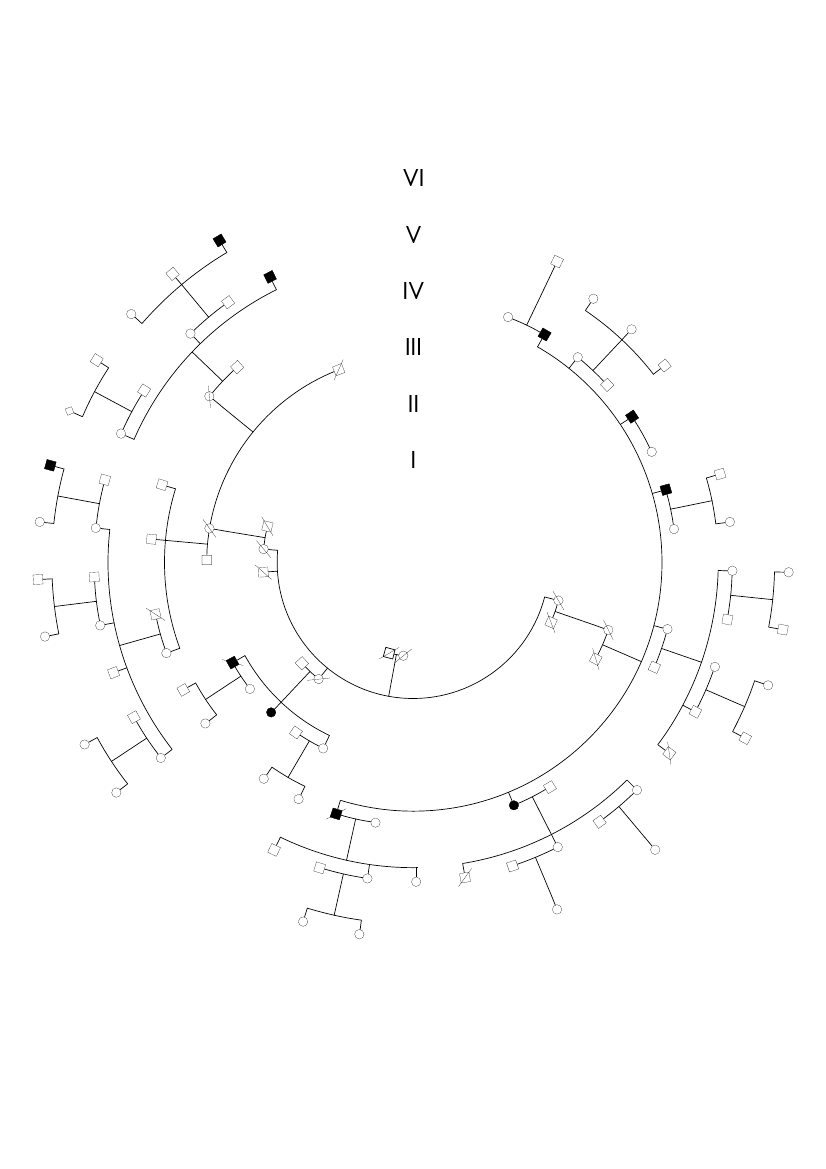


**Supplementary Figure 3.** Family 3 pedigree


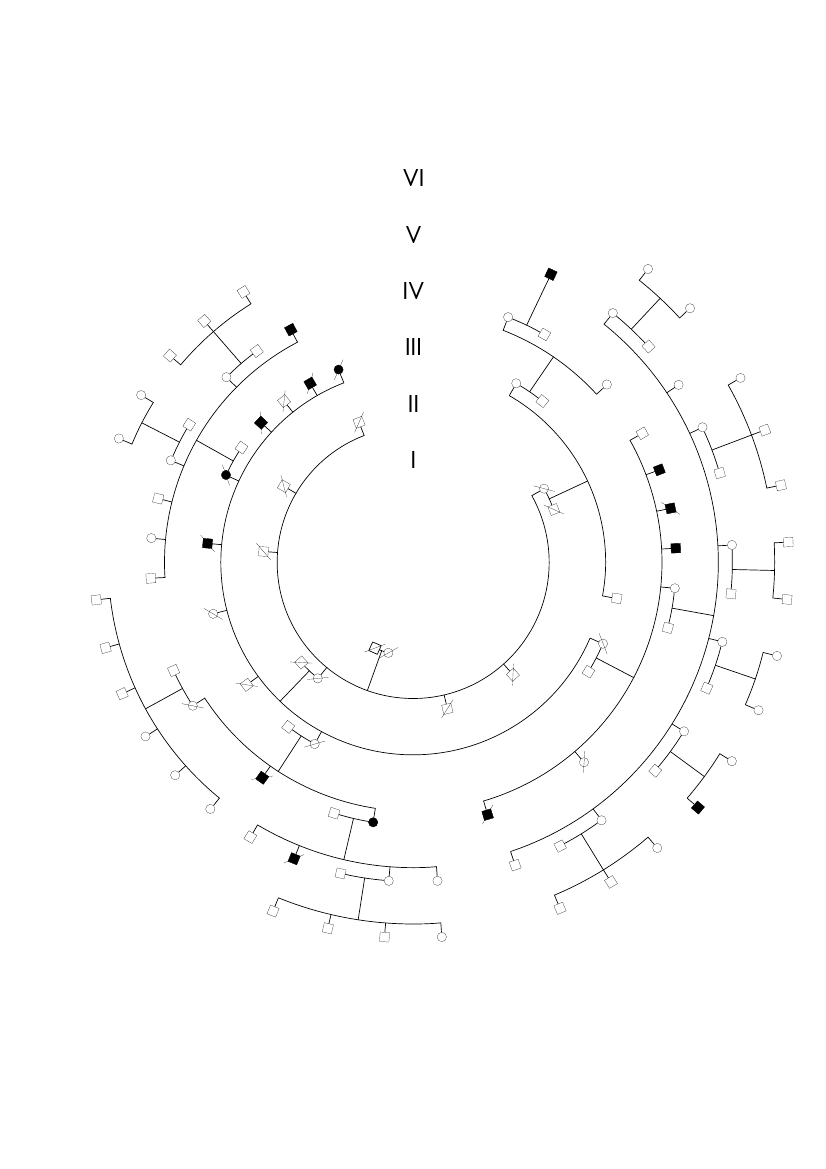


**Supplementary Figure 4.** Distribution of mtDNA *per cell* in each LHON family and in the Italian cohort, sorted by primary mutation.


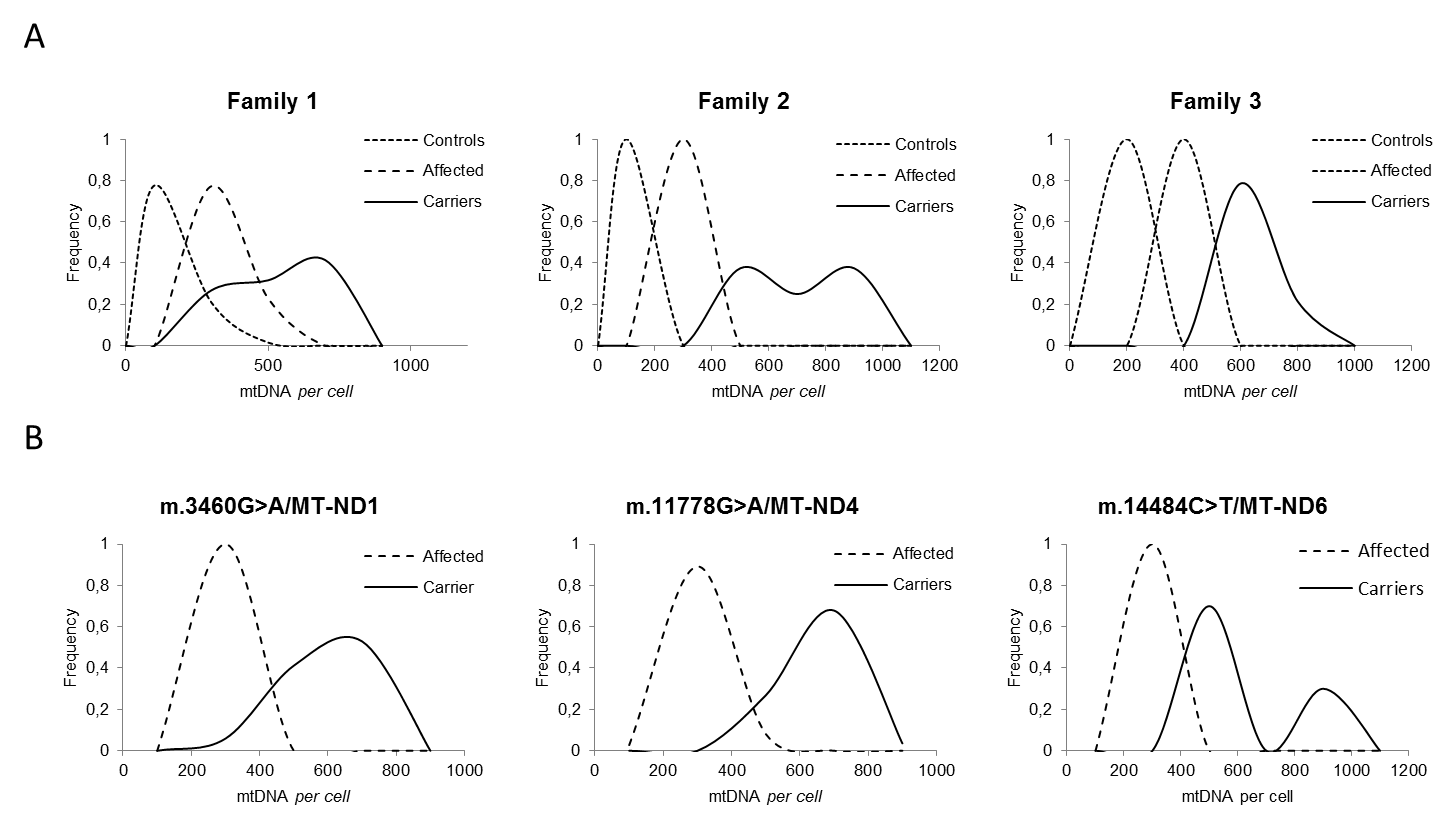


**Supplemetary Figure 5.**

(A) R output for the multiple regression analysis (best fitting model according to the R package BMA) performed on the following list of covariates: gender, age, retinal nerve fiber layer thickness (RNFL) measured in the temporal, superior, nasal and inferior peripapillary quadrants (QUADRANT T, QUADRANT S, QUADRANT N, QUADRANT I), RNFL average, RNFL minimum; disc area and macular thickness in the temporal-superior (TEMPSUP), superior (SUP), nasal-superior (NASSUP), nasal-inferior (NASINF), inferior (INF) and temporal-inferior (TEMPINF) quadrants. (B) Schematic representation of the retinal nerve fibers (light blue lines) coursing from the macula to the optic nerve head as evaluated by OCT (red perimacular sectors and blue peripapillary quadrants). Asterisks indicate the sectors that were inversely correlated with mtDNA content in blood cells. Thus, swelling of the fibers (increase of fiber thickness) in the sectors belonging to the papillo-macular bundle significantly correlated with reduced mtDNA content in blood cells.

A


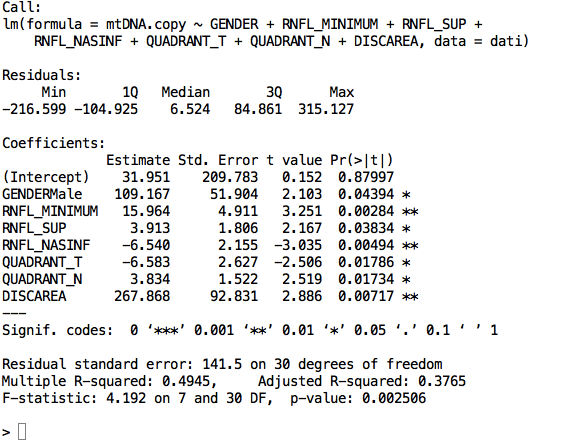


**OCT**

B


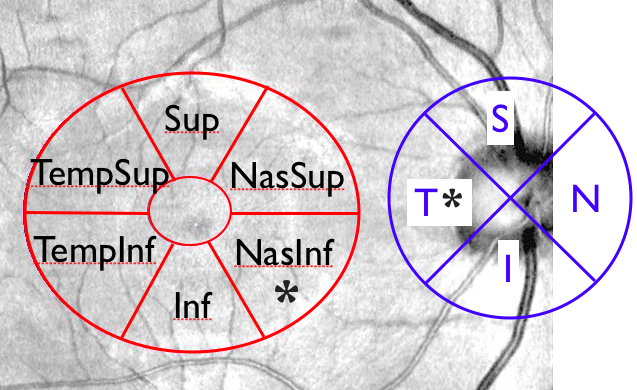


**Supplemetary Figure 6.** Scatter plot of mtDNA *per cell* with mean±SD sorted by mtDNA haplogroup, in affected (A) and in carriers (B), and sorted by mtDNA haplogroup J vs others, in affected (C) and in carriers (D), in the Italian cohort.


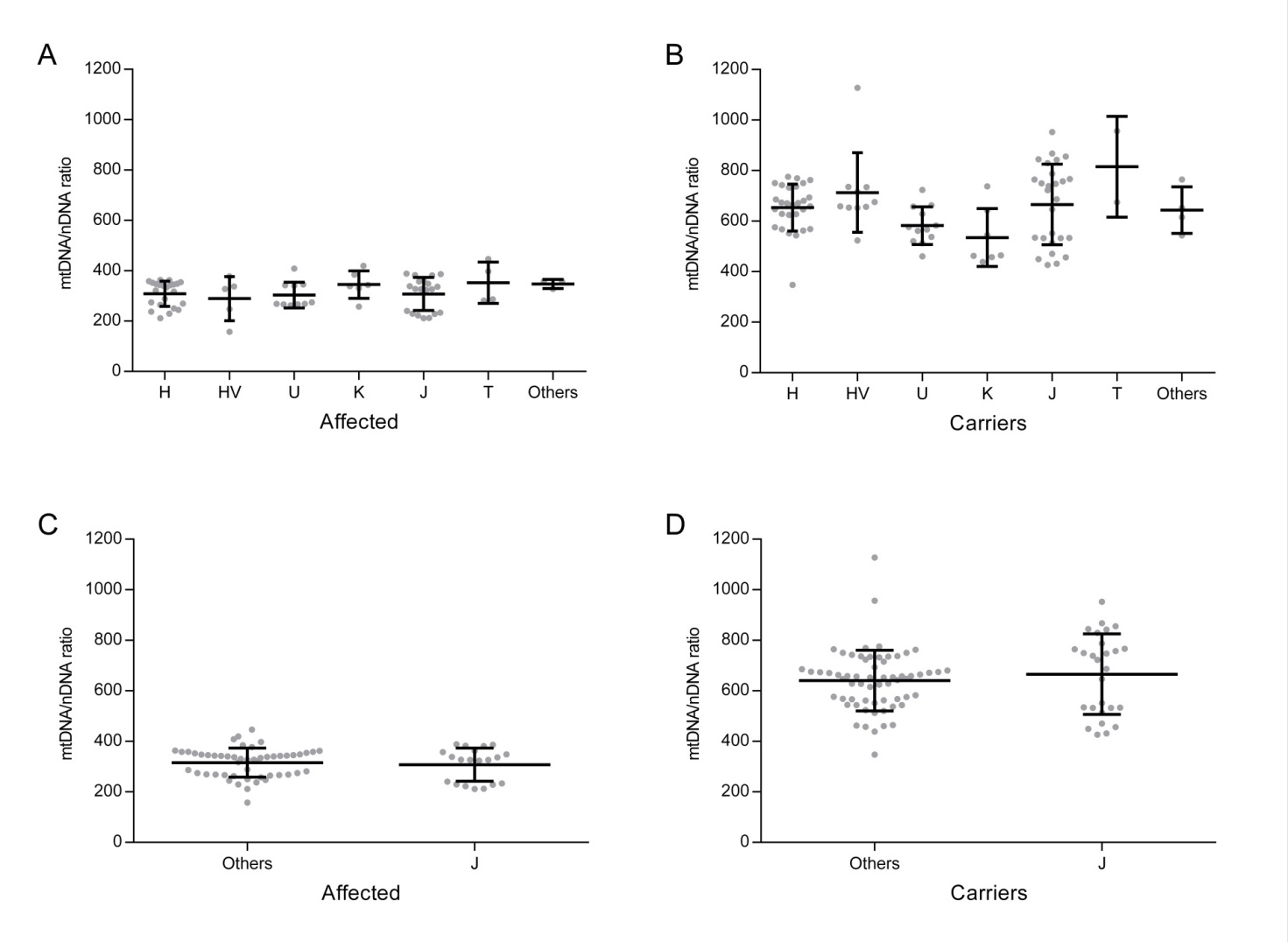


**Supplemetary Figure 7.** mtDNA *per cell* (mean±SD) sorted by PARL SNPs genotyping. Asterisks indicate statistical significance (p<0.05).


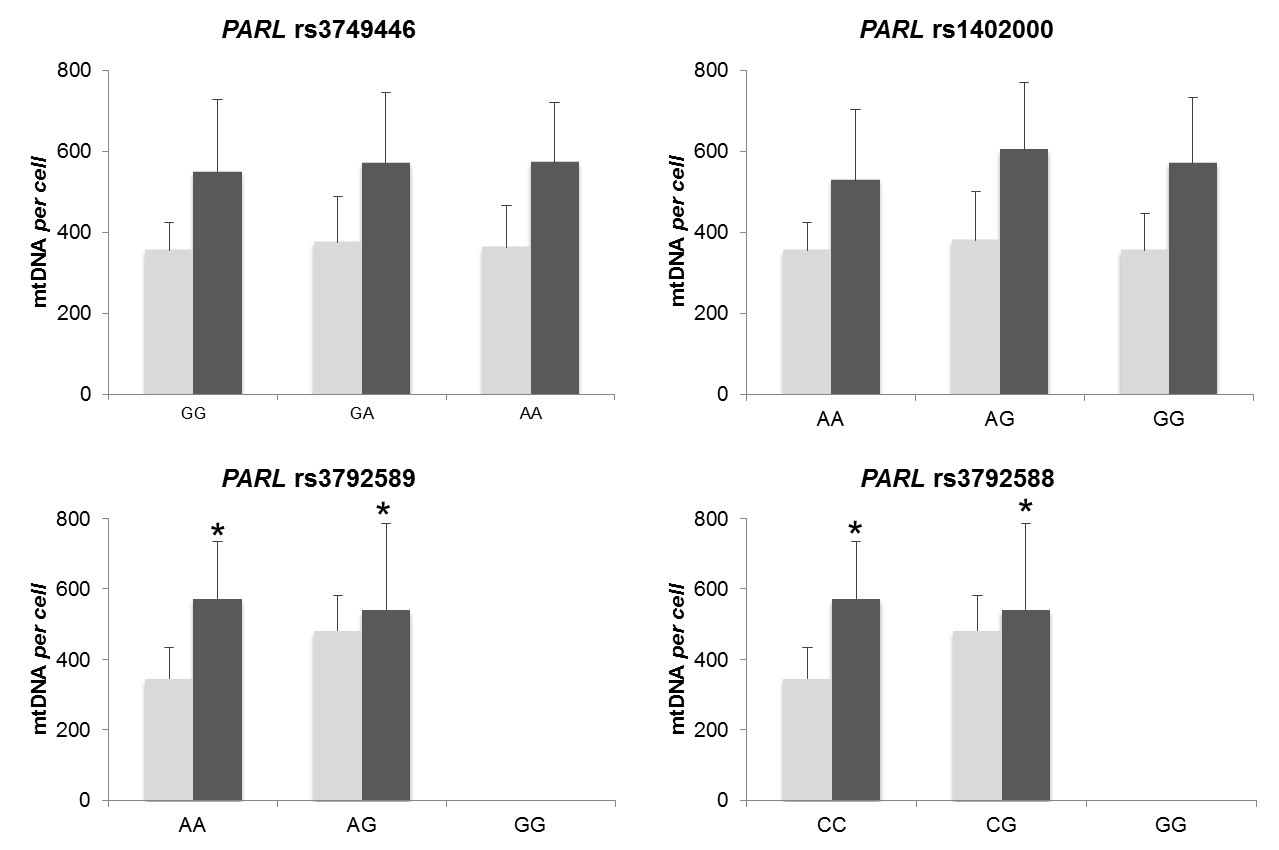


Affected Carriers
